# Potential Impact of PI3K-AKT Signaling Pathway Genes, KLF-14, MDM4, miRNAs 27a, miRNA-196a Genetic Alterations in the Predisposition and Progression of Breast Cancer Patients

**DOI:** 10.3390/cancers15041281

**Published:** 2023-02-17

**Authors:** Othman R. Alzahrani, Rashid Mir, Hanan E. Alatwi, Yousef M. Hawsawi, Amnah A. Alharbi, Abdulrahman H. Alessa, Elham Saleh Albalawi, Imadeldin Elfaki, Yousef Alalawi, Laila Moharam, Sabah H. El-Ghaiesh

**Affiliations:** 1 Department of Biology, Faculty of Science, University of Tabuk, Tabuk 47512, Saudi Arabia; 2 Genome and Biotechnology Unit, Faculty of Science, University of Tabuk, Tabuk 47512, Saudi Arabia; 3 Department of Medical Lab Technology, Faculty of Applied Medical Sciences, University of Tabuk, Tabuk 47512, Saudi Arabia; 4 College of Medicine, Al-Faisal University, Takhasusi Road, P.O. Box 50927, Riyadh 11533, Saudi Arabia; 5 Department of Biochemistry, Faculty of Science, University of Tabuk, Tabuk 47512, Saudi Arabia; 6 Department of Pathology, Faculty of Medicine, University of Tabuk, Tabuk 47512, Saudi Arabia; 7 Department of Surgery, King Salman Armed Forces Hospital, Tabuk 47512, Saudi Arabia; 8 Department of Pathology, King Salman Armed Forces Hospital, Tabuk 47512, Saudi Arabia; 9 Department of Pharmacology, Faculty of Medicine, University of Tabuk, Tabuk 47512, Saudi Arabia

**Keywords:** breast cancer, amplification refractory mutation system (ARMS), whole-exome sequencing (WES), signaling pathway gene (PI3K-AKT), Krüppel-like factor 14 gene, Mouse double minute 4 (MDM4) gene, microRNA (miR-27a; miR-196a)

## Abstract

**Simple Summary:**

The genomic landscape of breast cancer (BC) is complex. Previous research studies have not extensively elucidated the correlation of genotypes and allele variations of the PI3K, AKT-1, KLF-14, MDM4 and miRNAs 27a, miR-196a genes with the predisposition of Breast cancer in Saudi Arabia. Therefore, to cover this area of research, we conducted a case-control study on 230 subjects (115 cases and 115 controls). Genotyping was studied by using the ARMS-PCR and results were confirmed by Sanger sequencing. The novel and known gene variants were studied by Whole-exome sequencing using Illumina NovaSeq 6000 platform. Strong association was reported between the PI3K-AKT signaling pathway genes and KLF 14-AA, MDM4-GA, miR27a-GG and miR-196a-CT gene variants with the breast cancer susceptibility and progression. The results could help to classify and identify those at risk for Breast cancer in the future. WES for provide insight toward disease mechanisms for the development of more effective therapies.

**Abstract:**

Genome-wide association studies have reported link between SNPs and risk of breast cancer. This study investigated the association of the selected gene variants by predicting them as possible target genes. Molecular technique advances with the availability of whole-exome sequencing (WES), now offer opportunities for simultaneous investigations of many genes. The experimental protocol for PI3K, AKT-1, KLF-14, MDM4, miRNAs 27a, and miR-196a genotyping was done by ARMS-PCR and sanger sequencing. The novel and known gene variants were studied by Whole-exome sequencing using Illumina NovaSeq 6000 platform. This case control study reports significant association between BC patients, healthy controls with the polymorphic variants of PI3K C > T, AKT-1 G > A KLF 14 C > T, MDM4 A > G, miR-27a A > G, miR-196a-2 C > T genes (*p* < 0.05). MDM4 A > G genotypes were strongly associated with BC predisposition with OR 2.08 & 2.15, *p* < 0.05) in codominant and dominant models respectively. MDM4 A allele show the same effective (OR1.76, *p* < 0.05) whereas it remains protective in recessive model for BC risk. AKT1G > A genotypes were strongly associated with the BC susceptibility in all genetic models whereas PI3K C > T genotypes were associated with breast cancer predisposition in recessive model OR 6.96. Polymorphic variants of KLF-14 A > G, MDM4G > A, MiR-27aA >G, miR-196a-C > T were strongly associated with stage, tamoxifen treatment. Risk variants have been reported by whole exome sequencing in our BC patients. It was concluded that a strong association between the PI3K-AKT signaling pathway gene variants with the breast cancer susceptibility and progression. Similarly, KLF 14-AA, MDM4-GA, miR27a-GG and miR-196a-CT gene variants were associated with the higher risk probability of BC and were strongly correlated with staging of the BC patients. This study also reported Low, novel, and intermediate-genetic-risk variants of PI3K, AKT-1, MDM4G & KLF-14 by utilizing whole-exome sequencing. These variants should be further investigated in larger cohorts’ studies.

## 1. Introduction

Over the past years, despite the coronavirus disease (COVID-19) pandemic, the prevalence of breast cancer cases has raised dramatically and is ranked as the 2nd major cause of cancer-related death worldwide [1]. About 287,850 new breast cancer cases with more than 43,250 breast cancer-related deaths is expecting in USA in 2022 [1]. “Breast cancer is ranked as the first leading cause of cancer-related deaths in Saudi Arabia”. In 2020, the WHO estimated more than 3954 new breast cancer cases with over 1095 breast cancer-related deaths [2,3]. It has been revealed a substantial discrepancy in breast cancer incidence between Saudis and non-Saudis, which is likely due to gene expression and lifestyle differences [4,5]. Breast cancer is distinguished by its unique genetic, molecular, and clinical heterogeneity [6,7]. Recently molecular profiling has determined that 26% of hereditary tumors are caused by mutations in highly penetrant *genes* (*BRCA2, PTEN, BRCA1, TP53, CDH1*), which carry a lifelong risk of about 80% for breast cancer development [8].

**Figure 1:**
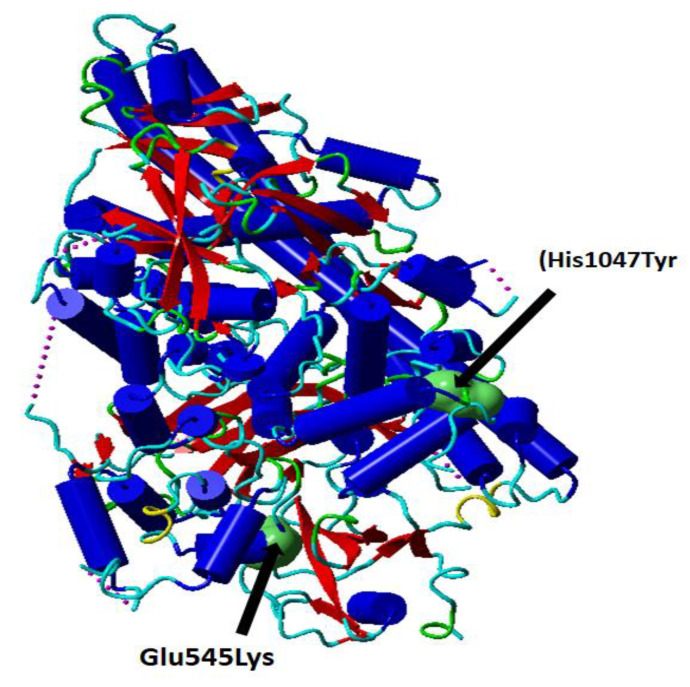
Cartoon presentation of the catalytic subunit of PI3K secondary structure PDB ID: 5XGH. The single nucleotide variations rs104886003 G > A (Glu545Lys) and rs121913281 C > T (His1047Tyr) are shown in green surface presentation. This figure (Figure 1) is prepared using YASARA, and modified from Elfaki et al. [30].

The *PI3K-AKT-mTOR* signaling pathway plays a significant role in the susceptibility of breast carcinoma [9]. Several *PI3K-AKT-mTOR* inhibitors have been developed for targeting the components of PI3K-AKT-mTOR pathway and promising therapeutic effects has been reported in breast cancer [10]. Some combination therapy with mTOR inhibitor and exemestane has significantly prolonged the overall survival (OS) of breast cancer patients with hormone receptor (HR)-positive metastatic breast cancer [11]. Therefore, targeting the three components of *PI3K-AKT-mTOR* signaling pathway has become promising therapeutic modalities in the treatment of breast cancer. With the advent of novel promising *PI3K-AKT-mTOR* pathway inhibitors, it is essential to identify the specific patients who can benefit from these *PI3K-AKT-mTOR* inhibitors. Next-generation sequencing (NGS) technology has made it possible to identify such genetic aberrations quickly and accurately. Many research studies have reported that breast cancer patients with *PIK3CA* mutations (Figure 1) have a better prognosis [12], while Sobhani et al. reported a worse prognosis for women with *PIK3CA*-mutant breast tumors [13]. Gerratana et al. studied 88 cases of metastatic breast cancer by multivariate logistic regression and reported that the detection of *PIK3CA* mutation in circulating tumor DNA was associated with lung metastasis (OR 3.74) [14].

*MDM4* (*MDM4* Regulator of P53) is a Protein Coding gene. [15]. The human MDM4 gene, which plays a role in apoptosis, encodes a 490-amino acid protein containing a RING finger domain and a putative nuclear localization signal. These oncogenic capabilities are regularly accomplished through *MDM4* gene amplification. However, several mutations in *MDM4* were reported to be associated with cancers such as breast cancer, endometrial cancer, and stomach cancer [16]. In breast malignancy, several SNPs, and mutations in *MDM4* were reported to promote breast growth including the rs11801299 G > A polymorphisms. *MDM4* rs11801299 G > A polymorphisms has been reported to be connected with retinoblastoma susceptibility [17], risk of gastric cancer in Chinese population [18], and risk of breast cancer [19]. In 2018, an Iranian study reported an association between the *MDM4* rs11801299 G > A polymorphisms and the susceptibility to breast tumor [20].

The Krüppel-like transcription factors (KLF) are a family of 17 transcription factors which are involved in the modulation of several genes that are essential for different cellular processes [21]. Recently, the importance of *KLF14*, also called *BTEB5*, was postulated in different genetic studies revealing its significant role as a primary gene expression regulator [22]. *KLF 14* has been shown to be a novel tumour suppressor and is often downregulated in human cancers, demonstrating its role as an important biomarker for disease progression and for developing new cancers treatments [23,24]. Meta-analysis and genome wide studies examining the effect of polymorphic variants found that rs972283 polymorphism in *KLF14* has high risk of developing diseases with the G allele associated with *T2D*, metabolic disorders in different populations and in another study with A allele which is associated with polycystic ovary syndrome in specific populations [25,26,27]. For *T2D* and breast cancer susceptible groups with the *KLF-14*- rs972283 variant association found to be weak among European and African population [28]. However, this variant is highly attributed to patient with genetic predisposition for T2D and with breast or prostate cancer [29]. Breast cancer frequently experiences oncogenic activation of the phosphatidylinositol-3-kinase (PI3K), protein kinase B (PKB/AKT), and mammalian target of rapamycin (mTOR) pathway, which promotes tumor development, disease progression, and therapy resistance. Recent studies suggest that the intricate interactions between the *PI3K-AKT-mTOR* pathway and several interacting cell signaling cascades can enhance the advancement of Breast cancer. The PIK3R1 gene encodes the PIK3R1 protein. PI3K is an important protein in the Akt signaling pathway which play important role in cell survival, differentiation, growth, glucose trafficking, and utilization. Glu545Lys (rs104886003); His1047Tyr (rs121913281) mutations induce confirmation change in the phosphatidylinositol- 4,5-bisphosphate 3-kinase catalytic subunit alpha (PIK3CA) protein (Figure 1).

MicroRNAs are small, single stranded, non-coding RNAs. Most of them are down expressed in cancer cells which guides to cellular transformation followed by tumor development and cancer progression. The miR-27a-3p is a single stranded, non-coding RNAs which is recognized as an oncogenic RNA in multiple malignancies including colorectal, Breast and gastric cancer. MiR-27a is mostly located in exosomes of the BC cancer cells [30,31,32]. It is reported that miR-27a-3p (exosomal) foster immune attach by activating PD-L1 via *MAGI2/PTEN/PI3K* axis in breast cancer [33]. The miR-27a rs895819 A/G is a common SNP that is found in the loop of the pre-miRNA and may have a key role in mi-R27a maturation [34,35]. The minimum free energy (MFE) is affected when there is a variation from A to G in this SNP, and as a result, the function of the miRNA might be affected to some extent. Several reported studies showed the possibility of this variant allele in decreasing the risk of cancer, while however other studies showed the opposite [36]. In one of the meta-analysis, miR-27a rs895819 gene polymorphism has been reported to be linked with the breast cancer susceptibility among Caucasians. Besides AA genotype of miR-27a conferred strong link with breast cancer susceptibility and AG genotype (heterozygous) as well as G-allele were protective factors [37].

Recent studies have shown that miR-196b acts as a tumor suppressor in various cancer types [38] in HepG2 cells that suppressed cell proliferation and induced apoptosis [39]. One of the most common SNP reported is *miR-196a*-rs11614913 C to T which may increase or decrease the extent of translation of the target protein therefore may alter the expression as well as the functions which in turn can increase cancer susceptibility [40]. MiR-196a has been reported to target several genes that may be involved in cell cycle, apoptosis, and differentiation. *MiR-196a* targets annexin-*A1* (*ANXA1*) gene which controls the physiological mechanisms such as exocytosis, hormone secretion, apoptosis, and signal transduction [41]. Some studies have been performed to establish the link between *miR-196a* (rs11614913) polymorphism and breast cancer risk [42]. Some studies report it as a protective factor [43] whereas others like *Omrani* et al. and Qi et al. indicated strong association with the breast cancer susceptibility [44,45].

The human genome’s protein-coding regions, which contain around 85% of the disease-causing variations, can almost entirely be covered by whole-exome sequencing (WES) [46]. The exome makes up around 1% of the entire human genome, making WES an incredibly potent tool for medical genetic research. Future case/control and family based NGS research will be more effective thanks to WES, which is frequently more affordable and permits the sequencing of more individuals [47]. More recently, 65 loci strongly linked with breast cancer were found by a genome-wide association study [48].

## 2. Materials and Methods

This study was conducted on primary BC patients (*n* = 115) and gender matched healthy women (*n* = 115) with no history of any type of cancer and who were not related to the patients from the general population. Formalin fixed paraffin embedded (FFPE) tissue specimens were obtained from the Division of Histopathology, King Salman Armed Forces Hospital North-western Region at Tabuk city and other hospitals. This study handled patient samples and records in accordance with the Declaration of Helsinki revised in 2013 under the ethical approval of Armed Forces Hospital Research Ethics Committee (KSAFH-REC-2020-345/8 September 2020) and the ethics committee of the University of Tabuk (protocol code UT-115-13-2020/24 April 2020). All patients obtained written informed consent form. The study experiment was carried out at Genome and Biotechnology Unit, Faculty of Science, University of Tabuk.

**Inclusion criteria of patients:**

The study included clinically confirmed cases of breast cancer patients who were Saudi women. Specimens were collected from primary breast cancer patients who had been diagnosed based on the clinical, histopathological, and radiological findings. The study also included breast cancer cases who had received chemotherapy, hormonal therapy, and radiotherapy.

**Exclusion criteria:**

The exclusion criteria included (1). Breast cancer patients diagnosed with multiple cancer types, (2). Patients who were unable to cope with the study protocol, (3). Non-Saudi women with breast cancer, (4). Any breast cancer patient with a history of previous significant malignancy.

**Inclusion criteria for healthy controls**

A healthy control cohort was prepared from the participants visiting for routine checkup to King Fahd Special Hospital, Tabuk, Saudi Arabia. These participants completed the informed consent form and filled questionnaire. The inclusion criteria included (1) Gender matched healthy women, (2) Women with age equal or greater than 40 years (≥40), (3) Only ethnic Saudi women.

**Exclusion criteria for healthy controls**

The exclusion criteria included healthy women with a family history of breast cancer, non-ethnic Saudi women, women with age below 40 years of age.

### 2.1. Demographic Data

Each breast cancer patient filled out a standardized questionnaire about their demographics, family history, and previous knowledge. To determine relevant clinical history, detailed laboratory and clinical data were collected.

### 2.2. DNA Extraction from Cases and Controls

The FFPE samples were collected from the pathology department and some peripheral blood specimens were also obtained by venipuncture and placed in EDTA tubes after assessing the clinicopathological findings. According to the manufacturer’s instructions, DNA was extracted from the specimens (FFPE) using the QIAamp DNA FFPE Tissue Kit (Cat-56404) from Qiagen (Hilden, Germany). Similarly, blood specimens were processed for genomic DNA extraction using the DNeasy Blood Kit (Cat-Noc69506) from Qiagen (Germany). The extracted DNA was dissolved in water devoid of nucleases and kept at 4 °C until needed.

The genomic DNA quality and integrity were checked using NanoDropTM (Thermo Scientific, Waltham, MA, USA), and the extracted DNA’s quality was investigated optically using the A260 nm/A280 nm ratio (1.83–1.99).

**Genotyping of PI3K rs121913281 C > T, MDM4 rs11801299 A > G, AKT-1 rs1130233 G > A, KLF-14, MDM4, miRNAs 27a and miR-196a**

The genotyping of the PI3K rs121913281 C > T, AKT-1 rs1130233 G > A, KLF 14-rs972283C > T, MDM4-rs11801299 A > G, miRNAs 27a -rs895819A > G and miR-196a-2 -rs11614913 C > T genes were determined using ARMS primers that were used elsewhere [20,26]. The ARMS primers for KLF-14, MDM4, miRNAs 27a and miR-196a genotyping are depicted in Table 1.

### 2.3. Preparation PCR Mix

The PCR reaction was carried out in a total of reaction volume of 12 μL, which was composed of the four primers FO (0.10 μL), RO (0.10 μL), FI (0.10 μL), RI (0.10 μL), (25 pmol of each primer), and 6 μL of green PCR Master Mix (2X) (Cat M712C) (Promega, Madison, WI, USA). The final volume of 12 μL was achieved by using nuclease-free ddH_2_O. Template DNA (50 ng) was added at the end. The thermocycling procedures were as follows: initial denaturation at 95 °C for 8 min, followed by 32 cycles of denaturation at 95 °C for 33 s; annealing for 35 s at 60 °C for AKT-1 (58 °C), PI3K (59 °C), KLF 14 (56.7 °C) for MDM4 (63 °C) for miRNAs 27a, (61 °C) for miR-196a-2; extension at 72 °C for 40 s, then final extension at 72 °C for 09 min.

### 2.4. Gel Electrophoresis and PCR Product Visualization

The PCR-amplified products were separated on 2% agarose gel electrophoresis, stained with sybre safe dye, and visualized under UV transilluminator from Bio-Rad, Hercules, CA, USA.

***AKT-1 rs1130233 G* > A gene variation**: The AKT-1 outer region was amplified by the outer primers FO and RO, yielding a band of 466 bp that served as a DNA purity check. A band of 213 bp was produced by the primers FI and RO amplifying the G allele, and a band of 298 bp was produced by the primers FI and RO amplifying the A allele (Supplementary Figure S1).

**Phosphatidylinositol 3-kinase (PI3K) rs121913281 C > T gene variation:** The two forward primers and common reverse primer sequence are shown in (Table 1). Primers F2T and common reverse primers amplify the (TT genotype), that is, the mutant-type allele (364 bp). Primers F1C and the common reverse primer generated a band (364 bp) for the wild type allele (CC genotype) as depicted in Supplementary Figure S2.

**Figure 2:**
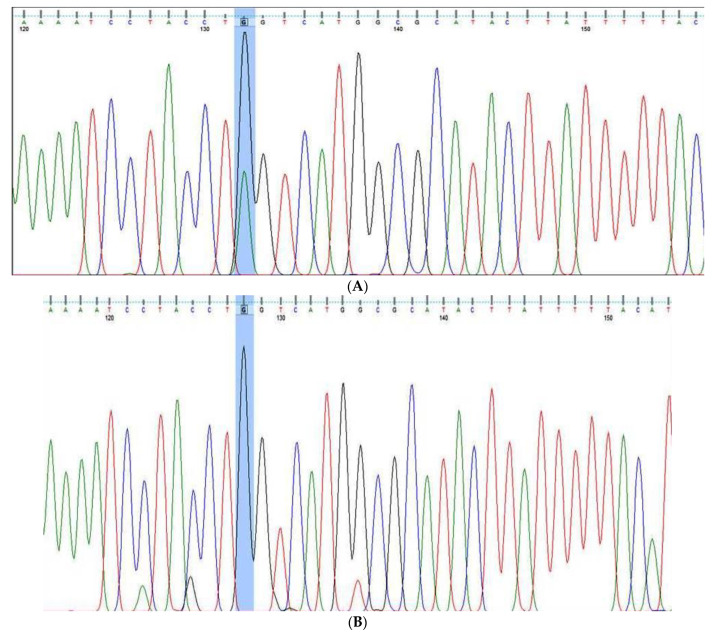

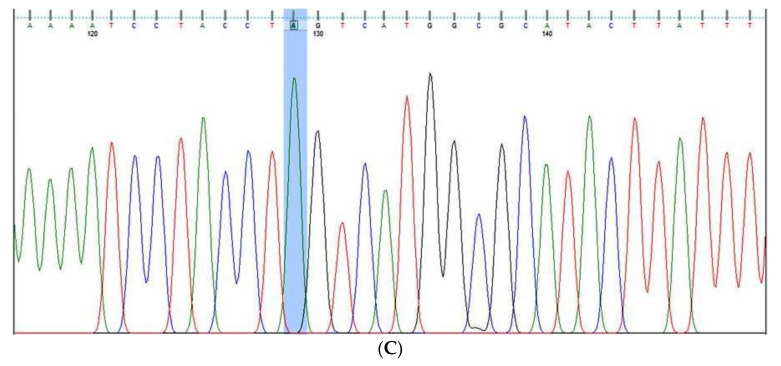
KLF-14 rs972283 G > A genotyping by Sanger sequencing. (**A**): Sanger sequencing for KLF-14 rs972283 genotyping (G/A alleles), (**B**): Sanger sequencing for KLF-14 rs972283 genotyping (G allele); (**C**): Sanger sequencing for KLF-14 rs972283 genotyping (A allele).

***KLF-14 rs972283 G >* A gene variation**: The KLF14′s outer region was amplified by the outer primers F1 and R1, producing a band of 437 base pairs that serves as a DNA purity control. A band of 221 base pairs (bp) was produced by primers F1 and R2 amplifying the A allele, and a band of 274 bp was produced by primers F2 and R1 amplifying the G allele. Results were confirmed by Sanger sequencing (Figure 2A–C).

**Figure 3:**
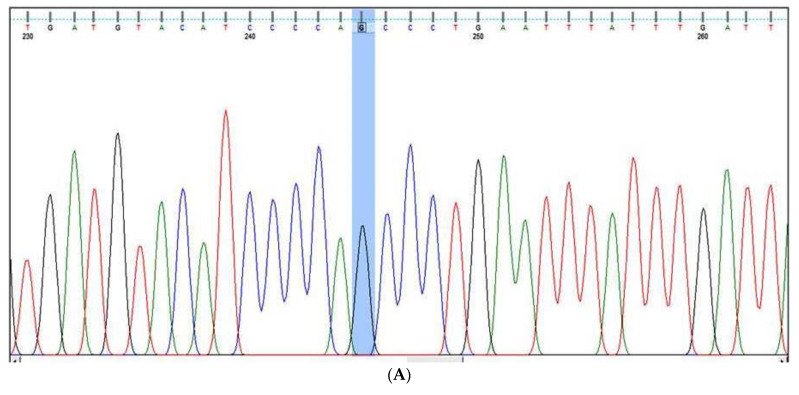

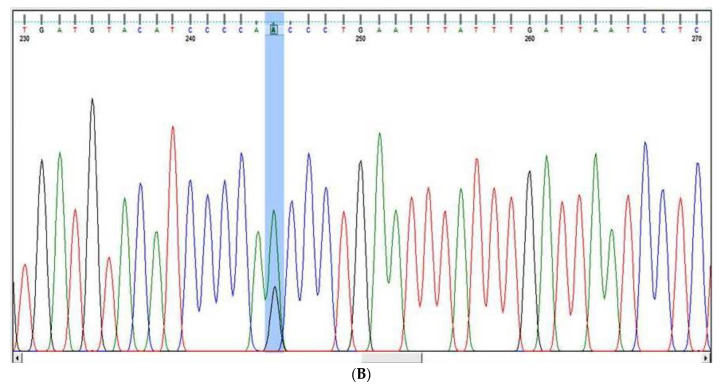
*MDM4* rs11801299 A > G genotyping by Sanger sequencing. (**A**,**B**) Sanger sequencing for MDM4 rs11801299 gene polymorphism (G allele) and AG genotype.

***MDM4* rs11801299 A > G gene variation:** The exterior primers A band of 468 bp was produced after Fo and R0 amplified the MDM4′s outer region, serving as a DNA purity checker. A band of 223 bp was produced by the A allele by primers FI and Ro, and a band of 301 bp was produced by the G allele by primers Fo and RI (as depicted in Supplementary Figure S3). The results were confirmed by Sanger sequencing as depicted in Figure 3A,B.

***MiR-27a rs895819 A > G* gene variation:** The miR-27′s outer region was amplified by the outer primers FO and RO, yielding a band of 353 bp that served as a DNA purity check. A band of 226 bp was produced by primers FI and R2 amplifying the A allele, and a band of 184 bp was produced by primers F2 and R1 amplifying the G allele (as depicted in Supplementary Figure S4).

***MiR-196a2 rs11614913 C > T* gene variation:** The miR-196a2 exon was flanked by primers FO and RO, which resulted in a 297 bp band that served as a DNA purity check. A band of 153 base pairs (bp) was produced by primers FO and RI amplifying the C allele, and a band of 199 bp was produced by primers FI and RO amplifying the T allele (as depicted in Supplementary Figure S5).

### 2.5. Sanger Sequencing for the Confirmation of Genotyping Results

To confirm the genotyping results of KLF-14 rs972283 G > A, MDM4 rs11801299 A > G, PI3K-rs121913281 C > T AKT-1 rs1130233 G > A, and miR-196a2 rs11614913 C > T detected by ARMS-PCR, 20 randomly selected PCR products from the PCR systems for polymorphic sites in these gene were sequenced using Sanger sequencing. Two primers F seq and R Seq were used as sequencing primers as depicted in Table 1 for the detection of the genotyping in the above genes. The PCR amplification was done followed by purification using QIAquick PCR Purification Kit from Qiagen (Germany). Finally, the purified PCR products were sequenced by Applied Biosystems sequencer.

### 2.6. Whole Exome Sequencing

For whole exome sequencing, DNA was extracted from peripheral blood using standard Qiagen nucleic acid isolation kits. The library was prepared as per the instruction manual of the Twist 2.0 Exome kit and sequencing was performed using the Illumina NovaSeq 6000 platform as per the user manual. The sequencing reads QC was carried out using FastQC v0.11.9. Raw reads were filtered to remove sequencing adapters and low-quality bases using TrimGalore v0.6.6 software. High quality (HQ) reads thus obtained were mapped on the hg38 human reference genome; variant calling (single nucleotide variants (SNVs), small InDels) was done with the Genome Analysis Toolkit (GATK) v4.2.4.1 software best practice pipeline using haplotype caller.

Variant annotation was carried out using different databases and tools. The RefSeq database was used for identification and characterization of genes associated variants. The disease association for variants was derived using databases like OMIM and ClinVar. The population frequency information from 1000 genomes, ExAC, GnomAD exome, GnomAD genome, and ESP, was used for elimination of common variants/polymorphism. For prediction of implication of coding non-synonymous SNVs on the structure and function of protein, PolyPhen-2 and the SIFT score was used. Furthermore, all variants were separately analyzed by multiple prediction tools for in-silicon variant effect prediction. All variants were then interpreted based on the ACMG guidelines (PMID:25741868) and variants classified as pathogenic, likely pathogenic, and variant of uncertain significance were reported. Some results are presented in the Supplementary Tables S1–S3.

### 2.7. Statistical Analysis

All statistical analyses were determined using Med-Calc software, version 20.027 (medcalc.org/calc/odds_ratio.php)/SPSS 16.0 (SPSS, Inc., Chicago, IL, USA) as well as statistical software version 9.4 (SAS Institute, Inc., Cary, NC, USA) and Stata statistical software (StataCorp. 2013. Release 13. College Station, TX, USA). Deflection from Hardy–Weinberg disequilibrium (HWD) was determined by Chi-square (χ^2^) ‘goodness of fit test’. A *p*-value < 0.05 is observed as statistically significant difference. The distribution and association of PI3K rs121913281 C > T, AKT-1 rs1130233 G > A, KLF 14 (rs972283 C > T), MDM4 rs11801299 A > G, miRNAs 27a rs895819A > G, and miR-196a-2 rs11614913 C > T alleles and genotypes between the groups was determined by Chi-square test. To assess the association between the risk of BC and the genotypes of KLF 14 (rs972283 C > T), MDM4 (rs11801299 A > G), miRNAs 27a (rs895819A > G), and miR-196a-2 (rs11614913 C > T), we generated odds ratios (ORs), risk ratios (RRs), and risk differences (RDs) with 95% confidence intervals (CIs). The OR was calculated by dividing the probabilities in the first group by the odds in the second group.

## 3. Results

### 3.1. Demographic Features of BC Patients

Table 2 provides an overview of the demographic characteristics of the 115 breast cancer patients who were treated in succession. Complete clinical information was available for 100/115 breast cancer cases. However, BC patients were divided into two groups according to their age, with those over 40 (*n* = 75, 65.2%) and those under 40 (*n* = 40, 34.8%). Of them, 30/100 (30%) BC cases were in early stage (stage I and II) and 70/100 (70%) cases were in advanced stage (stages III and IV) of breast cancer. The BC cases accounted for 70 (70%) cases: 10 (10%), 30 (30%), and 60 (60%) of BC were, respectively, in grades I, II, and III according to the histological classification. According to the receptor status, 60 patients (60%) were positive for the estrogen receptor, 70 patients (70%), and 43 patients (43%), respectively, for the progesterone receptor, estrogen receptor, and the Her2/neu receptor. A total of 75 (75%) of the patients had distant metastases, compared to 25 (25%) of the patients who did not.

### 3.2. Biochemical Characteristics of Healthy Controls and Breast Cancer Patients

As reported in Table 2, the age at inclusion was comparable in both the patient and control groups with the mean age of ~27 years. Sex hormone levels for progesterone follicle stimulating hormone, luteinizing hormone (LH), and testosterone were significantly different, whereas no significant difference was indicated for estradiol levels between the patients and controls. The serum lipid profile for HDL, LDL, total cholesterol, and triglycerides also showed significant differences between the patients and controls.

### 3.3. Hardy–Weinberg Equilibrium

There was no deviation from the Hardy–Weinberg equilibrium for the control group in the genotype distributions and allele frequencies of the SNPs located in the genes for four gene SNPs for KLF 14 (rs972283 C > T) gene polymorphism HWE is (χ^2^ = 070 *p <* 0.79), for MDM4 rs11801299 A > G HWE is (χ^2^ = 0.190 *p <* 0.66), for miRNAs 27a rs895819A > G HWE is (χ^2^ = 0.0358 *p <* 0.849) and for miR-196a-2 rs11614913 C > T HWE is (χ^2^ = 1.928 *p* < 0.164). As a result, we randomly selected 10% of the samples from the normal control group to review the genotyping results, demonstrating that the accuracy rate was greater than 99%.

### 3.4. Comparative Analysis of the PI3K rs121913281 C > T, AKT-1 rs1130233 G > A, KLF 14 (rs972283 C > T), MDM4 rs11801299 A > G, miRNAs 27a rs895819A > G and miR-196a-2 rs11614913 C > T Genotypes in Breast Cancer Patients and Gender Matched Controls

Breast cancer patients were more likely to have the frequencies of AKT-1 (rs1130233 G > A genotypes GG (50%), GA (40%), and AA (10%), compared to gender matched controls who were more likely to have frequencies of GG (78.43%), GA (19.60%), and AA (1.90%), respectively (Table 3). A statistically significant difference in the AKT-1 (rs1130233 G > A genotypes was seen between breast cancer patients and healthy controls (*p* < 0.0001). Additionally, it was discovered that breast cancer patients had a higher frequency of the A allele than healthy controls (0.30 vs. 0.12) (Table 3).

The frequencies of PI3K rs121913281 in breast cancer cases was CC (26.54%), CT (51.32%), and TT (22.12%), compared to gender matched controls who were more likely to have frequencies of CC (16.66%), CT (79.41%), and TT (3.92%), respectively (Table 3). A statistically significant difference in the PI3K C > T genotypes was seen between breast cancer patients and healthy controls (*p* < 0.0001). Additionally, it was discovered that breast cancer patients had a higher frequency of the T allele than healthy controls (0.48 vs. 0.43) (Table 3).

Similarly, the frequencies of KLF 14 rs972283 G > A genotypes in case were GG (29.95%), GA (39.13%), and AA (33.91%), compared to gender matched controls who were more likely to have frequencies of GG (46.86%), GA (45.21%), and AA (13.91%), respectively. A statistically significant difference in the KLF 14 rs972283 G > A genotypes was seen between breast cancer patients and healthy controls (*p* = 0.002). Additionally, it was discovered that breast cancer patients had a higher frequency of the A allele than healthy controls (0.53 vs. 0.37) (Table 3). The MDM4 rs11801299 G > A genotype frequency was GG (62.60%), GA (34.78%), and AA (2.60%) in breast cancer patients and controls, respectively, and GG (78.26%), GA (20.86%), and AA (0.86%) in controls. The MDM4′s rs11801299 G > A SNP was statistically significant (*p <* 0.03) between breast cancer patients and controls. Additionally, it was discovered that breast cancer patients had a higher frequency of the A allele than healthy controls (0.20 vs. 0.11) (Table 3).

The frequency of miRNAs 27a A > G genotypes in breast cancer cases and matched controls was AA (56.52%), GA (28.69%), and GG (14.78%), and controls AA (57.69%), GA (36.92%), and GG (5.38%), respectively. The miRNAs 27a rs895819 A > G gene polymorphism determined between breast cancer cases and matched controls was significant (*p* < 0.033). Additionally, it was discovered that breast cancer patients had a higher frequency of the G allele than healthy controls (0.29 vs. 0.24) (Table 3).

The frequency of miR-196a C > T genotypes in matched healthy and breast cancer patients was CC (60.86%), CT (38.26%), and TT (0.86%), and controls CC (74%), CT (22.22%), and TT (3.70%), respectively. The miR-196a T > C gene polymorphism determined between cases and matched controls was strongly associated (*p* < 0.010). Additionally, it was discovered that breast cancer patients had a higher frequency of the T allele than healthy controls (0.20 vs. 0.15) (Table 3).

### 3.5. Logistic Regression Analysis of AKT1 rs1130233 G > A Genotypes to Predict the Risk of Breast Cancer

Our findings indicated a strong association between the AKT1 rs1130233-GA genotype with the breast cancer susceptibility in the codominant model, with an OR = 3.20, (95%) CI = 1.6829–6.084, RR = 1.84, and *p* < 0.0004. Similarly, the AKT1 rs1130233-AA genotype was strongly linked to breast cancer susceptibility with an OR = 3.20, (95%) CI = 1.682–6.084, RR = 3.69, and *p* < 0.044. A strong association was reported between the *AKT 1*–GG and (*AKT 1*–GG + AA) genotypes with the breast cancer susceptibility with an OR = 3.63, (95%) CI = 1.969–6.715, RR = 2.01, and *p* < 0.0001 in the dominant inheritance model. A significant correlation was reported in the recessive inheritance model between *AKT-1*(GG + GA) and AKT1-AA genotypes and breast cancer susceptibility with an OR = 5.5, (95%) CI = 1.185–26.036, RR = 3.15, and *p* < 0.029 (Table 4). With an OR = 3.21, 95% CI = 1.906–5.41, RR = 1.96, and *p* = 0.0001, the AKT1-A allele was significantly associated with breast cancer susceptibility in allelic comparison.

### 3.6. Logistic Regression Analysis of PI3K rs121913281 C > T Genotypes to Predict the Risk of Breast Cancer

Our findings indicated a protective association between the PI3K rs121913281 CT genotype and breast cancer susceptibility in the codominant model, with an OR of 0.40 (95%) CI = 0.204–0.804, RR = 0.62 and *p* < 0.009 (Table 5), whereas the PI3K rs121913281 TT genotype was strongly linked to breast cancer susceptibility with an OR of 3.54, (95%) CI = 1.0544–11.896, RR = 2.62 and *p* < 0.040. A non-significant association was reported between the PI3K –CC and (PI3K –CT + TT) genotypes with the breast cancer susceptibility with an OR = 0.55, (95%) CI = 0.28–1.078, RR = 0.71 and *p* < 0.081 in the dominant inheritance model. A strong correlation was reported in the recessive inheritance model between the PI3K –(CC + CT) and PI3K –TT genotypes and breast cancer susceptibility with an OR = 6.96, (95%) CI = 2.33–20.78, RR = 3.81, and *p* < 0.0005 (Table 6). With an OR = 1.18, 95%CI = 0.80–1.73, RR = 1.09, and *p* = 0.387, the PI3K T allele was not associated with breast cancer susceptibility in allelic comparison.

### 3.7. Logistic Regression Analysis of KLF 14 rs972283 G > A Genotypes to Predict the Risk of Breast Cancer

To estimate the relationship between the KLF 14 rs972283 C > T genotypes and the risk of breast cancer, a multivariate analysis using a logistic regression calculated the odds ratio (OR) and the risk ratio (RR) with 95% confidence intervals (CI) for each group. The data are summarized in Table 6. Our findings demonstrated a strong association between the KLF 14 -AA genotype and increased breast cancer patient susceptibility in the codominant model, with an OR = 3.69 (CI = 1.7672–7.7282), RR = 2.07 (CI = 1.3204–3.2493), and *p* < 0.0005. While the KLF14-GA genotype was not linked to breast cancer susceptibility, with an OR = 1.31 (CI = 0.7171–2.4004), RR = 1.12 (CI = 0.8681–1.4554), and *p* < 0.37. In the dominant inheritance model, there is a significant association between the KLF14-GG and KLF14-(GA + AA) genotypes and leads to increased breast cancer susceptibility with OR = 1.87 (CI = 1.07–3.26), RR = 1.87 (CI = 1.0465–1.7336), and *p* < 0.026. In the recessive inheritance model, there is a strong correlation between the KLF14 (GG + GA) and KLF14-AA genotypes that increases breast cancer susceptibility OR = 3.17 (CI = 1.6507–6.1076), RR = 1.94 (CI = 1.2618–2.9971), and *p* < 0.0005 (Table 6). In the context of allelic comparison, the KLF14-A allele was found to be strongly associated with breast cancer susceptibility, as indicated by an OR = 1.99 (CI = 1.3759–2.9015), RR = 1.42 (CI = 1.1693–1.7295), and *p* < 0.0003. In the over dominant inheritance model, there was no association observed between the KLF14 GA and KLF14-AA + GG genotypes; OR = 1.28 (CI = 0.7599–2.1694), RR = 1.13 (CI = 0.8747–1.4643), and *p* < 0.350 (Table 6).

### 3.8. Logistic Regression Analysis of MDM4 rs11801299 G > A Genotypes to Predict the Risk of Breast Cancer

Our findings showed a strong association between the MDM4-GA genotype and increased breast cancer patient susceptibility in the codominant model, with OR = 2.08 (CI = 1.1509–3.771), RR = 1.48 (1.0492–2.0918), and *p* = 0.015. While the MDM4-AA genotype had an OR = 3.07 (CI = 0.3819–36.8217), RR = 2.22 (0.4048–12.2007), and *p* = 0.35, indicating that it was not linked to breast cancer susceptibility. The dominant inheritance model showed a strong association between the MDM4-GG and (GA + AA) genotypes, which increases breast cancer susceptibility (OR = 2.15 (CI = 1.2010–3.8487), RR = 1.51 (1.0747–2.1247), and *p* = 0.010) (Table 7). In the recessive inheritance model, there was a significant protective effect between the MDM4 (GG + GA) and MDM4 -AA genotypes that reduced breast cancer susceptibility (OR = 0.46 (CI = 0.2598–0.8326), RR = 0.64 (0.4561–0.9060), and *p* = 0.010) (Table 7). With an OR = 1.76, (CI = 1.0249–3.0542), RR = 1.38 (0.9921–1.9324), and *p* = 0.040, the MDM4-A allele was significantly associated with breast cancer susceptibility in allelic comparison. In the over dominant inheritance model, there was a protective effect between the MDM4-GA and MDM4-AA/GG genotypes with an OR = 0.49 (CI = 0.2738–0.8932), RR = 0.68 (0.4844–0.9660), and *p* = 0.019 (Table 7).

### 3.9. Logistic Regression Analysis of miRNAs 27a rs895819 A > G Genotypes to Predict the Risk of Breast Cancer

Our findings showed a strong association between the miR27a-GG genotype and increased breast cancer patient susceptibility in the codominant model, with an OR = 2.80 (CI = 1.0937–7.1793), RR = 1.83 (0.9663–3.4913), and *p* = 0.031. In contrast, the MDM4-AG genotype had an OR = 0.79 (CI = 0.4559–1.3803), RR = 0.90 (0.7129–1.1463), and *p* = 0.41, indicating that it was not linked to breast cancer susceptibility. There was no association observed between the miR27a-GG and miR27a-(GA + GG) genotypes in the dominant inheritance model with an OR = 1.26 (CI = 0.7645–2.1072), RR = 1.12 (0.8735–1.4489), and *p* < 0.35 (Table 8). In the dominant inheritance model, there was no association between the miR27a-GG and miR27a-(GA + GG) genotypes, with OR = 1.26 (CI = 0.7645–2.1072), RR = 1.12 (0.8735–1.4489), and *p* = 0.35 (Table 8). With an OR = 1.31 (CI = 0.8774–1.9638), RR = 1.15 (0.9344–1.4351), and *p* = 0.18, the miR27a-A allele was not linked to breast cancer susceptibility. In the over dominant inheritance model, there was no difference between the miR27a-AG and miR27a-AA/GG genotypes, with an OR = 1.45 (CI = 0.8488–2.4926), RR = 1.18 (0.9354–1.5017), and *p* = 0.019 (Table 8).

### 3.10. Logistic Regression Analysis of miR-196a-2 rs11614913 C > T Genotypes to Predict the Risk of Breast Cancer

Our findings showed that the miR-196a-CT genotype was strongly associated with an increased risk of developing breast cancer in the codominant model, with OR = 2.02 (CI = 1.2021–3.651), RR = 1.45 (1.0714–1.965), and *p* = 0.009 (Table 9). While the miR-196a-2-TT genotype had a OR = 0.28 (CI = 0.032–2.49), RR = 0.70 (0.483–1.03), and *p* = 0.25, none of these traits were related to breast cancer susceptibility. The dominant inheritance model showed a strong association between the miR-196a-2-CC and miR-196a-(CT + TT) genotypes, which increases BC susceptibility (OR = 1.83 (CI = 1.07–3.26), RR = 1.34 (1.017–1.776), and *p =* 0.026) (Table 9).

In the recessive inheritance model, there was no correlation between the miR-196a-2-(CC + CT) and miR-196a-TT genotypes, with OR = 0.22 (CI = 0.0263–1.981), RR = 0.60 (0.4387–0.931), and *p* = 0.18 (Table 9). With an OR = 1.43 (CI = 0.9021–2.290), RR = 1.19 (0.937–1.522), and *p* = 0.12, the miR-196a-T allele was not linked to breast cancer susceptibility in allelic comparison (Table 9).

### 3.11. Correlation KLF-14 rs972283 A > G Genotypes with Clinicopathological Features of Cases

The Krüpple-like Transcription Factor KLF-14 rs972283 G SNP was not related to age status in BC (*p* = 0.83) (Table 10); however, a strong association was reported with respect to breast cancer staging (*p* = 0.006). Similarly, a strong association was reported with respect to the estrogen receptor status (*p* = 0.002), the progesterone receptor status (*p* = 0.027), and the Her2/neu receptor status (*p* < 0.02). However, the histological grade of BC was not correlated (*p* = 0.90) with the KLF-14 rs972283 A > G SNP (Table 10). The Her2/neu receptor status (*p* = 0.02), the progesterone receptor status (*p* = 0.027), and the KLF-14 rs972283 A > G SNP all showed significant correlations. Additionally, the KLF-14 rs972283 A > G SNP was significantly associated with the status of receiving either herceptin (*p* = 0.020) or tamoxifen (*p* = 0.002) treatment.

### 3.12. Correlation of MDM4 rs11801299 G > A with Clinical Features of the Cases

The MDM4 rs11801299 G > A SNP was not associated with age status in BC (*p <* 0.46) (Table 11). A strong association was indicated with the staging of BC patients (*p <* 0.0005). While the MDM4 rs11801299 G > A SNP was correlated with the histological grade of BC (*p <* 0.03) (Table 11), it was significantly associated with the Her2/neu receptor status (*p* < 0.008) but not associated with ER status (*p <* 0.38) or PR status (*p <* 0.49). In addition, the MDM4 rs11801299 G > A SNP was associated with herceptin treatment (*p* = 0.020), but not with the tamoxifen treatment status (*p* = 0.47).

### 3.13. Correlation of miRNAs 27a rs895819 A > G Genotypes with Clinical Features of the Cases

Age status in BC was significantly correlated with the MiR-27a rs895819 A > G SNP (*p* < 0.0005) (Table 12). The stage of BC was significantly correlated with the MiR-27a rs895819 A > G SNP (*p* = 0.0006); however, the histological grade of BC was not linked to the MiR-27a rs895819 A > G SNP (*p =* 0.87) (Table 12). The status of progesterone and Her2/neu receptors were not significantly correlated with the MiR-27a rs895819 A > G SNP (*p* = 0.55 and *p* = 0.07, respectively). Furthermore, the MiR-27a rs895819 A >G SNP was not associated with the presence of a tamoxifen treatment (*p* = 0.090) but was associated with herceptin treatment (*p* = 0.005).

### 3.14. Correlation of miR-196a-2 rs11614913 C > T Genotypes with Clinical Features of Cases

The MiR-196aC > T variation was not correlated with age status in BC (*p* < 0.11) (Table 13). The miR-196aC > T SNP was significantly correlated with the staging of BC (*p =* 0.007). However, the miR-196a C > T polymorphism was not correlated with the histological grade of BC patients (*p* < 0.78) (Table 13). Regarding receptor status, the miR-196a C > T SNP was significantly associated with the Her2/neu receptor status (*p* < 0.006) but not with estrogen receptor status (*p* = 0.41) and progesterone receptor status (*p* = 0.305). In addition, the miR-196a-2 rs11614913 C > T SNP was associated with herceptin treatment (*p* = 0.006) but not with the tamoxifen treatment status (*p* = 0.39). The MiR-196a C > T SNP was strongly associated with the distant metastasis status in BC patients (*p* < 0.017 (Table 13).

### 3.15. Disease Progression with Respect to Mutation or Gene Polymorphisms

**Figure 4:**
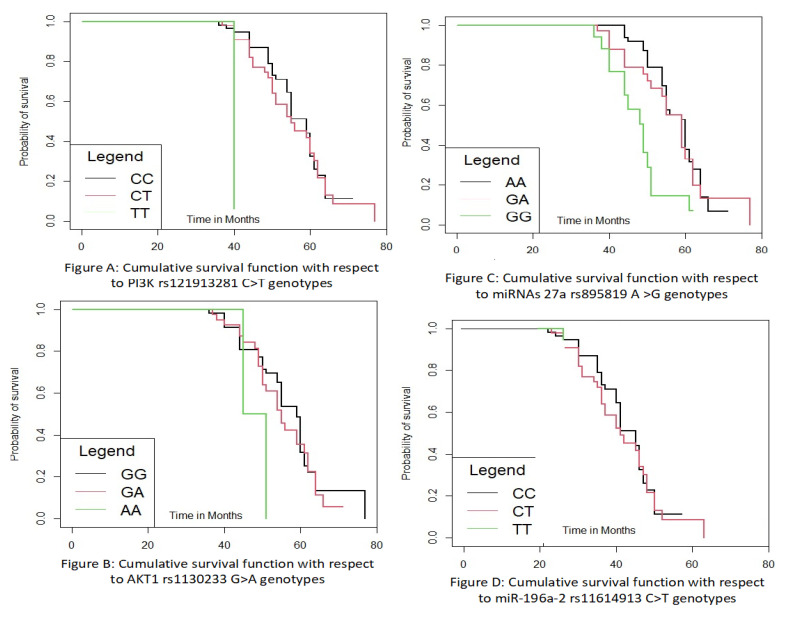
The figures represent the cumulative and overall survival by the Kaplan–Meier method, log-rank test, and Cox regression method of breast cancer patients according to gene polymorphism PI3K rs121913281 C > T (**A**), AKT1 rs1130233 G > A (**B**), miRNAs 27a rs895819 A > G (**C**), and miR-196a-2 rs11614913 C > T (**D**) polymorphism, log-rank test (*p* < 0.05).

Overall, due to the increasing survival rates of breast cancer patients, longer follow-up studies are required to shed light on prognostic data. In our study, there were statistically significant differences in disease-free survival according to the gene polymorphisms (*p* > 0.05). There was a statistically significant difference in OS (overall survival) at 6 years according with some selected gene variants like PI3K rs121913281 C > T, AKT1 rs1130233 G > A, miRNAs 27a rs895819 A > G, miR-196a-2 rs11614913 C > T with lower survival in patients with polymorphic genotypes (*p* < 0.05) and with HR (hazard ratio) of above 2.30. This difference was independent of adjuvant treatment with endocrine therapy. The figures has shown the cumulative and overall survival by the Kaplan–Meier method, log-rank test, and Cox regression method of breast cancer patients according to gene polymorphism. The Kaplan–Meier curve method with respect to PI3K rs121913281 C > T gene polymorphism is depicted in Figure 4A, For AKT1 rs1130233 G > A gene polymorphism is shown in Figure 4B, for miRNAs 27a rs895819 A > G gene polymorphism is depicted in Figure 4C, and for the miR-196a-2 rs11614913 C > T gene polymorphism is Figure 4D, log-rank test (*p* < 0.05). The genotypes did not influence patient age, stage at diagnosis, or tumor grade.

**Whole exome sequencing in breast cancer patients:**

The most common AKT1 gene variants identified by whole exome sequencing in all our breast cancer cases were AKT-1 rs1130233 C > T, c.726G > A, p.Glu242, and p.E242 (Supplementary Table S1). The most common BRCA1 gene variants identified by whole exome sequencing in all our breast cancer cases were BRCA1 rs799917 C > T/c.2612C > T/p.Pro871Leu, BRCA1 *rs1799966 T* > *C/c.4900A* > *G/p.Ser1634Gly/p.S1634G*, BRCA1 rs1799949 G > A, c.2082C > T/p.Ser694/p.S694, BRCA1 rs1060915 A > G/c.4308T > C/p.Ser1436/p.S1436, and BRCA1 rs16940 A > G/c.2311T > C/p.Leu771/p.L771 (Supplementary Table S2). Similarly, the most common variants identified in the KLF14 gene were rs111400400, rs184537657, and rs111731678; two of them were missense variants (KLF14 rs111400400, rs184537657) and one was a silent or synonymous variant (rs111731678) (Supplementary Table S3). Similarly, the most common mutation reported in the PIK3 gene was rs4344416 G/A, a silent synonymous variant. We have identified the most common mutation in the PIK3 gene as rs4344416 G/A, a silent synonymous variant.

## 4. Discussion

Breast cancer (BC) is the most common cancer in women and one of the important death causes in women all over the world in including Saudi Arabia. In this study we have investigated if some SNPs of differences in the PI3K-AKT signalling pathway genes, KLF 14 (rs972283 C > T), MDM4 rs11801299 A > G, miRNAs 27a rs895819A > G and miR-196a-2 rs11614913 C > T genes affect the pathogenesis in Saudi population.

### 4.1. Comparative Analysis of Phosphoinositide 3-Kinase (PI3K)/AKT Pathway in Breast Cancer

Phosphoinositide 3-kinase (PI3K)/AKT/mTOR pathway gene variations are typically discovered in breast tumours and are linked to cellular change, carcinogenesis, cancer progression, and medication resistance. Breast cancer tumour tissue reveals AKT1 and MTOR mutations [49,50]. Therefore, studying genetic polymorphisms in the mTOR pathway may shed light on connections between obesity and breast cancer risk. The mTOR pathway may be crucial in the development of breast cancer. Few studies have looked at the relationship between cellular factors that are common in breast cancers and the mTOR pathway’s genetic variation and breast cancer risk, and subtypes and only a small number of single-nucleotide polymorphisms (SNPs) have been examined [51,52].

AKT is also called protein kinase B. It is a serine-threonine kinase that functions as a mediator of PI3K-Akt-mTOR signaling pathway. AKT has important role in an array of cellular processes. Many single nucleotide polymorphisms (SNP) in *AKT* gene have been observed to be associated with various types of cancers including breast cancer [53,54]. Our findings reported a strong association between the AKT1 rs1130233-GA genotype with the breast cancer susceptibility in the codominant model, with an OR of 3.20 (95%) CI = (1.6829 to 6.084), RR = 1.84 and *p*< 0.0004 and AKT1 rs1130233-AA genotype was strongly linked to breast cancer susceptibility with an OR of 3.20 (95%) CI = (1.682 to 6.084), RR = 3.69 and *p* < 0.044. (Table 4).

Studies have reported that PI3K-AKT-mTOR pathway can be associated in the development of Brain or central nervous system (CNS) metastasis from breast cancer patients [55]. PI3KR1-rs706716 has been reported to be strongly associated with CNS metastasis in metastatic breast cancer patients and may be included in a predictive composite score to detect early Brain metastasis irrespective of breast cancer subtype. Similarly, PI3K rs121913281 C > T showed a strong correlation between the recessive inheritance model and breast cancer susceptibility with an OR = 6.96 and for TT gene in codominant model with OR 3.54. A strong correlation was reported in the recessive inheritance model between PI3K (CC + CT) and PI3K –TT genotypes and breast cancer susceptibility with an OR = 6.96, (95%) CI ((2.33- to 20.78), RR = 3.81, and *p* < 0.0005) (Table 5). The PI3K/AKT/mTOR and RAF/MEK/ERK pathways have been indicated to be activated by mutations and chromosomal translocation in the vital targets. Research studies has shown that PI3K/AKT/mTOR signaling pathway are dysregulated in most of the malignancies including breast carcinogenesis [56]

### 4.2. Comparative Analysis of KLF 14, MDM4 in Breast Cancer

KLF 14 messenger RNA is significantly decreased in many types of human malignancies, such as breast cancer and colorectal carcinoma [57,58]. The frequencies of MDM4 rs11801299 G > A and KLF-14 rs972283 A > G genotypes determined in the cases of breast cancer and gender matched healthy controls (Table 3).Results showed that the AA genotype of the KLF-14-rs972283 A > G was associated with BC (Table 3), Moreover, the results indicated that the KLF-14 (rs972283 A > G) SNP genotype was significantly different in cases at early sate and cases at advance stage (Table 10). One of the study reveal that KLF14 reduction serves as a mechanism leading to centrosome amplification and tumorigenesis and further indicated that KLF14 serves as a tumour suppressor and reported its potential as biomarker and therapeutic target for several malignancies [23,59]. KLF14 inhibits the progression of cervical cancer by targeting ITGB1 via the PI3K/AKT signalling pathway [60]. In one of the studies reported that the whole body loss of KLF14 function in male mice does not result in metabolic abnormalities as assessed under chow and HFD conditions and concluded that there is redundancy for the role of KLF14 in the mouse and a diverging function in human malignancies [61]. It was reported that KLF14/miR-1283 signaling is downregulated cell proliferation in HER2+ breast cancer. Also it was suggested that the KLF14/miR-1283/TFAP2C axis inhibited HER2+ breast cancer progression, which might provide novel insight into mechanical exploration for this disease [62]. Results also indicated that there was significant difference in the KLF-14 (rs972283 A > G) SNP genotype between cases with positive and cases with negative oestrogen receptor (Table 10). In addition, KLF-14 rs972283 A > G SNP was significantly associated with the tamoxifen and Herceptin treatment status (Table 10).

These results are consistent with the study reported that KLF-14 is downregulated in brain cancer and colorectal cancer [59,61]. In addition, the KLF14 suppresses the cell growth and transformation of the cell mediated by oncogene KRAS [50]. The down regulation of the KLF14 in cancers suggested that it has an anti-tumour role [58]. Moreover, loss of KLF leads to amplification of centrosome and tumour progression [57]. Recently, it was shown that KLF14 suppresses the cervical cancer progression by reduction of the cell proliferation and enhancement of the apoptosis through integrin β1 (ITGB1) via the PI3K/AKT signalling pathway [59,60]. KLF14 and the KLF-14 (rs972283 A > G) roles in the breast cancer development remain to be investigated in future studies.

MDM4 negatively regulates of P53 that imposes negative feedback loop, and the influence of the MDM4 gene variations on cancer development was studied by different groups [63]. Nevertheless, some studies conflicting results [63,64]. MDM4 suppresses the transcriptional activity of p53 inhibition, and with MDM2 regulate the degradation of p53. Gene variations in MDM4 were reported to be associated with risk to cancer [65,66]. MDM4 rs11801299 G > A was associated with breast cancer predisposition in this study (Table 3 and Table 7). The MDM4 rs11801299 GA genotype and A allele were associated with increased risk to the breast cancer (Table 7). Results showed that the MDM4 rs11801299 G > A genotype was significantly different in cases in early and grade I stage and cases in advanced and grade II stage of breast cancer (Table 11). Furthermore, the MDM4 rs11801299 G > A SNP genotype distribution was associated with the Herceptin treatment status (Table 11). These results are inconsistent with the result of some previous studies reported that the MDM4 rs11801299 SNP is not associated with cancer [19,67,68]. However, our result may be in partial agreement with a study reported that the rs11801299 was significantly associated with risk to retinoblastoma in Chinese population [69]. The suppression of the MDM4 has been suggested as a therapeutic strategy for the reactivation of the p53 in hepatoblastoma [69]. The role of the SNPs on the structure and the functions of the MDM4 remain to be elucidated in further protein-protein interaction studies [69,70].

### 4.3. Comparative Analysis miRNAs 27a and miR-196a

The GG genotype of the miRNAs 27a rs895819 A > G was associated with risk to breast cancer in this study (Table 3). MiR-27a rs895819 A > G SNP was strongly associated with cases age status in BC and the staging of BC (Table 12). Moreover, MiR- 27a rs895819 A >G SNP was associated with the Herceptin treatment (Table 12). Previous studies to investigate the possible association of rs895819 SNP with the risk of breast cancer have been conducted in different populations [71]. The result however did not lead to a clear conclusion on whether there is a clear association. Yang et al., 2010 [72] suggested the protective role of the G allele rs895819 in breast cancer risk in the German populations However, this protective role of this SNP was not found in the Italian population [68]. In the Chinese population, Zhang et al., 2012 [73] suggested that there was no association between this SNP and breast cancer risk. However, in another study also in the Chinese population the G allele of rs895819 was reported to confer a protective role in the younger population [74]. The miR-27a enhances proliferation of the tumor cell by inhibition the AKT or tyrosine signaling pathways [75]. More studies can be performed with increased sample sizes and in different ethnicities to better understand the association of this SNP with breast cancer. Our results indicated that the miR-196a-CT genotype of rs11614913 was strongly associated with increased breast cancer susceptibility (Table 3, Table 9 and Table 13). Results showed that the miR-196a-rs11614913 C > T SNP was associated with the staging of breast cancer and Her2/neu receptor status (Table 13). This result is consistent with previous studies that reported the association of this SNP with cancer [74]. The C allele of the miR-196ars11614913 has been reported to increase the expression of the miR-196a-2 [73,74]. Moreover, this result is also consistent with a study reported increased expression of miR-196a in breast cancer tissues in comparison to normal tissues and that this increased miR-196a-2expression is associated with the increased stage of the breast cancer [76,77]. Jiang et al. [78] has reported that the estrogen causes up regulation of miR-196a in breast cancer cells that are estrogen receptor positive. The miR-196a up regulation by estrogen enhances the development of breast cancer via targeting the SPRED1 [78]. Since the C allele of the miR-196a rs11614913 enhances the expression of the miR-196a [72]. Our results may be consistent with the previous study [78], despite the fact that there was no association with estrogen receptor status (Table 13). This may be due to the relatively limited sample size used in this study which is one of the limitations. The rapidly evolving technology of whole exome sequencing has made it possible to analyze genomic characteristics of tumor samples at an unprecedented speed.

### 4.4. Whole Exome Sequencing in Breast Cancer

Detecting pathogenic intronic variants resulting in aberrant splicing remains a challenge in routine genetic testing. Several whole exome sequencing studies reported frequently mutated genes in breast cancer such as PIK3CA (13–42%), TP53 (43–75%), ARID1A (10–21%), RB1 (11–16%) and PTEN (11–15%) BRCA2 (13–26%) [79]. WES is a promising technology for developing biomarkers to be used in the clinic to better select patients for specific therapies [46,48]. Similarly in case of our study the most common BRCA1 gene variants identified by whole exome sequencing in our breast cancer cases were BRCA1 rs799917 C > T/c.2612C > T/p.Pro871Leu and BRCA1 rs1799966 T > C/c.4900A > G/p.Ser1634Gly/p.S1634G, BRCA1 rs1799949 G > A, c.2082C > T/p.Ser694/p.S694, BRCA1 rs1060915 A > G/c.4308T > C/p.Ser1436/p.S1436 and BRCA1 rs16940 A > G/c.2311T > C/p.Leu771/p.L771. (Supplementary Tables S2 and S3) Whole-exome sequencing may improve the risk assessment and to provide insight toward disease mechanisms for the development of more effective therapies. These results encourage conducting further studies using a large sample size, using different ethnic groups, and using large clinical trials for developing targeted therapies that could benefit breast cancer patients.

## 5. Conclusions

It was concluded that a strong association between the PI3K-AKT signaling pathway gene variants with the breast cancer susceptibility and progression. Similarly, KLF 14–AA, MDM4-GA, miR27a-GG and miR-196a-CT gene variants were associated with the higher risk probability of BC and were strongly correlated with staging of the BC patients. This study also reported Low, novel, and intermediate-genetic-risk variants of PI3K, AKT-1, MDM4G & KLF-14 by utilizing whole-exome sequencing. These variants should be further investigated in larger cohorts’ studies. Whole-exome sequencing is critical for improved risk assessment and to provide insight toward disease mechanisms for the development of more effective therapies. These results encourage conducting further studies using a large sample size and in different populations.

## Figures and Tables

**Table 1:** Amplification-refractory mutation system PCR primers.

Gene	Allele	Sequence	Product Size	Annealing Tempt
**ARMS primers for AKT-1 rs1130233 G > A gene variation**				
AKT 1Fo		5′-GGCTACTCTCCATGGCACCAGAC-3′	466 bp	58 °C
AKT 1Ro		5′-GAGGTTCTCCAGCTAGGGGAAAGG-3′		
AKT 1 FI	(G allele)	5′-TGTTCTTCCACCTGTCCCGGTAG 3′	213 bp	
AKT 1 RI	(A allele)	5′-CCGGTCCTCGGAGAACACAAGT-3′	298 bp	
**Phosphatidylinositol 3-kinase (PI3K) rs121913281 C > T gene variation**				
PIK3 **C** (wild)	(C allele)	5′-TTTCATGAAACAAATGAATGATGCA**C**-3′	364 bp	59 °C
PIK3 **T** (mutant)	(T allele)	5′-TTTCATGAAACAAATGAATGATGCA**T**-3′	364 bp	
PIK3 common R		5′-TTCAAAGTTTACCTTTTTGGACTTAAGGC-3′		
**ARMS primers for *KLF-14 rs972283 G > A* gene variation**				
KLF14 Fo		5′-GTCATAGGTCAAACAGCTAGATATTGGGT-3′	437 bp	60 °C
KLF14 Ro		5′-TCTACAGGACCAACTCAAATTATGAGGT-3′		
KLF14 FI	(G allele)	5′-TCATTGTATACTTGGAAAAAATCCTACATG-3′	274 bp	
KLF14 RI	(A allele)	5′-TATGTAAAAATAAGTATGCGCCATGCCT-3′	221 bp	
**ARMS primers for *MDM4* rs11801299 A > G gene variation**				
MDM4 Fo		5’-TTACTGTGTTGCTCATGTTTGTCTAGA-3′	468 bp	56.6 °C
MDM4 Ro		5′-TTTGTCTATAAATTTGTTCTGACCATGA-3′		
MDM4 FI	(A allele)	5′-GTTAACCTTGTGATGTACATCCCAAA-3′	223 bp	
MDM4 RI	(G allele)	5′-TAAAGAGGATTAATCAAATAAATTCAGTGC-3′	301 bp	
**ARMS primers for miR27a G > A gene variation**				
miR27a Fo		5′-GGCTTGACCCCTGTTCCTGCTGAACT-3′	353 bp	63 °C
miR27a Ro		5′-TTGCTTCCTGTCACAAATCACATTGCCA-3′		
miR27a FI	(G allele)	5′-GGAACTTAGCCACTGTGAACACGACTTTGC-3′	184 bp	
miR27a RI	(A allele)	5′-CTTAGCTGCTTGTGAGCAGGGTCCCCA-3′	226 bp	
**ARMS primers for miR-196a2rs11614913 T > C gene variation**				
miR-196a2 Fo		5′-ACCCCCTTCCCTTCTCCTCCAGATAGAT-3′	297bp	61 °C
miR-196a2 Ro		5′-AAAGCAGGGTTCTCCAGACTTGTTCTGC-3′		
miR-196a2 FI	(T allele)	5′-AGTTTTGAACTCGGCAACAAGAAACGGT-3′	199bp	
miR-196a2 RI	(C allele)	5′-GACGAAAACCGACTGATGTAACTCCGG-3′	153bp	
**Sanger sequencing primers**				
**AKT1 rs1130233 A > G**				
AKT1-F		5′-CTGCTGTGGGGTGACTTGTTC-3′	272 bp	**60 °C**
AKT1-R		5′-CTGCTGTGGGGTGACTTGTTC-3′		
**KLF14 rs972283 G > A**				
KLF14-F		5′-CTCCTCCCCATTTCTCATCA-3′	297 bp	**59 °C**
KLF14-R		5′-CCAAGAAAATACAAAGAGGAAAGG-3′		
***MDM4* rs11801299 A > G**				
*MDM4-F*		5′-TTACTGTGTTGCTCATGTTTGTCTAGA-3′	468 bp	**58 °C**
*MDM4-R*		5′-TTTGTCTATAAATTTGTTCTGACCATGA-3′		
**PI3K rs121913281 C > T**				
PI3K-F		5′-CTGGAATGCCAGAACTACAATC-3′	182 bp	**61 °C**
PI3K-R		5′-GTTCAATGCATGCTGTTTAATTG-3′		

**Table 2:** Baseline clinicopathological characteristics of BC patients.

Parameters		Total Patient No. = 115		*n* (%)	
Age		Age > 40 year		75 (65%)	
		Age ≤ 40 year		40 (35%)	
Association with BC stage		Early (I and II)		30 (30%)	
		Advanced (III and IV)		70 (70%)	
Histopathological grades		Grade I		10 (10%)	
		Grade II		30 (30%)	
		Grade III		60 (60%)	
Estrogen receptor status		Positive		60 (60%)	
		Negative		40 (40%)	
Progesterone receptor status		Positive		70 (70%)	
		Negative		30 (30%)	
Her2/neu status		Positive		43 (43%)	
		Negative		57 (57%)	
Distant metastasis status		Positive		75 (75%)	
		Negative		25 (25%)	
Association with herceptin treatment		Herceptin		32 (32%)	
		No Herceptin		68 (68%)	
Association with tamoxifen treatment		Tamoxifen		62 (62%)	
		No Tamoxifen		38 (38%)	
Biochemical characteristics of healthy controls and breast cancer patients.					
Characteristic	Controls ^a^		Cases ^a^		*p* ^b^
Age at inclusion in study ^c^	40.01 ± 2.62		38.06 ±6.70		0.229
Progesterone (pmol/L) ^d^	16.46 (3.20–19.80)		19.95 (1.98–35.90)		<0.001
Testosterone levels (ng/dL) ^d^	17.19 (9.15–38.68)		60.37 (42.17–82.46)		<0.001
Estradiol levels (pmol/L) ^d^	240.89 (142.76–470.14)		250.20 (179.90–501.15)		0.158
LH levels (mIU/mL) ^d^	0.09 (0.08–1.49)		3.90 (0.98–9.28)		<0.001
FSH levels (mIU/mL) ^d^	0.42 (0.46–3.66)		5.77 (3.29–6.70)		<0.001
Fasting glucose (mmol/L) ^c^	5.69 ± 0.43		5.90 ± 0.32		<0.063
Triglycerides (mmol/L) ^c^	1.98 ± 0.77		2.19 ± 0.97		<0.060
Cholesterol (mmol/L) ^c^	1.58 ± 0.28		1.49 ± 0.37		<0.050

^a^ Total of 115 breast cancer cases and 130 control subjects were included. ^b^ Student’s *t*-test for continuous variables (variables with normal distribution), Mann–Whitney U test (variables that were not normally distributed). ^c^ Values are presented as mean ± standard deviation. ^d^ Values presented as median (interquartile range).

**Table 3:** Association of PI3K rs121913281 C > T, AKT-1 rs1130233 G > A, KLF 14 (rs972283 C > T), MDM4 rs11801299 A > G, miRNAs 27a rs895819A > G, and miR-196a-2 rs11614913 C > T genotypes between breast cancer patients and gender matched controls.

Comparisons of the AKT1 rs1130233 G > A genotypes between cases and gender matched healthy controls									
Subjects	*n*	GG	GA	AA	Df	X^2^	G	A	*p* value
Cases	100	50 (50%)	40 (40%)	10 (%)	2	18.91	0.70	0.30	0.0001
Controls	102	80 (78.43%)	20 (19.60%)	02 (1.90%)			0.88	0.12	
**Comparisons of the PI3K rs121913281 C > T genotypes between cases and gender matched healthy controls**									
Subjects	*n*	CC	CT	TT	Df	X^2^	C	T	*p* value
Cases	113	30 (26.54%)	58 (51.32%)	25 (22.12%)	2	22.1	0.52	0.48	0.0001
Controls	102	17 (16.66%)	81 (79.41%)	04 (3.92%)			0.57	0.43	
**Comparisons of the KLF 14 rs972283 G > A genotypes between cases and gender matched healthy controls**									
Subjects	*n*	GG	GA	AA	Df	X^2^	G	A	*p* value
Cases	115	31 (29.95%)	45 (39.13%)	39 (33.91%)	2	13.41	0.57	0.53	0.002
Controls	115	47 (46.86%)	52 (45.21%)	16 (13.91%)			0.63	0.37	
**Comparisons of the MDM4 rs11801299 G > A genotypes between cases and controls (*p* values)**									
Subjects	*n*	GG	GA	AA	Df	X^2^	G	A	*p* value
Cases	115	72 (62.60%)	40 (34.78%)	03 (2.60%)	2	7.0	0.80	0.20	0.030
Controls	115	90 (78.26%)	24 (20.86%)	01 (0.86%)			0.89	0.11	
**Comparisons of the miRNAs 27a rs895819 A >G genotypes between cases and gender matched healthy controls**									
Subjects	*n*	AA	GA	GG	Df	X^2^	A	G	*p* value
Cases	115	65 (56.52%)	33 (28.69%)	17 (14.78%)	2	6.77	0.71	0.29	0.033
Controls	130	75 (57.69%)	48 (36.92%)	07 (5.38%)			0.76	0.24	

**Table 4:** Association of AKT 1 rs1130233 G > A genotypes with Breast cancer cases.

Genotypes	Controls	Breast Cases	OR (95%CI)	Risk Ratio (RR)	*p* Value
	**102**	**100**			
**Codominant model**					
*AKT 1* –GG	80	50	**1(ref.)**	**1(ref.)**	
*AKT 1* –*GA*	20	40	3.20 (1.6829 to 6.084)	1.84 (1.2590 to 2.707)	0.0004
*AKT 1* –*AA*	02	10	3.20 (1.6829 to 6.084)	3.69 (1.0344 to 13.180)	0.044
**Dominant model**					
*AKT 1* –GG	80	50	**1(ref.)**	**1(ref.)**	
*AKT1* –(GG + AA)	22	50	3.63 (1.9690 to 6.715)	2.01 (1.3858 to 2.926)	0.0001
**Recessive model**					
*AKT1* –(GG + GA)	100	90	**1(ref.)**	**1(ref.)**	
*AKT 1* –*AA*	02	10	5.55 (1.1854 to 26.036)	3.15 (0.8848 to 11.2712)	0.029
**Allele**					
*AKT 1 –G*	180	140	**1(ref.)**	**1(ref.)**	
*AKT 1 –A*	24	60	3.21 (1.9064 to 5.419)	1.96 (1.3850 to 2.798)	0.0001
**Over dominant model**					
*AKT 1* *–GA*	20	40	**1(ref.)**	**1(ref.)**	
*AKT 1* *–GG/AA*	82	60	0.36 (0.1945 to 0.688)	0.57 (0.3930 to 0.847)	0.008

**Table 5:** Association of PI3K rs121913281 C > T genotypes with breast cancer cases.

Genotypes	Controls	Breast Cases	OR (95% CI)	Risk Ratio (RR)	*p* Value
	**102**	**113**			
**Codominant model**					
PI3K –CC	17	30	**1 (ref.)**	**1 (ref.)**	
PI3K –CT	81	58	0.40 (0.2048 to 0.804)	0.62 (0.4140 to 0.9306)	0.009
PI3K –TT	04	25	3.54 (1.0544 to 11.896)	2.62 (0.9783 to 7.029)	0.040
**Dominant model**					
PI3K –CC	17	30	**1 (ref.)**	**1 (ref.)**	
PI3K –(CT–TT)	85	83	0.55 (0.2839 to 1.078)	0.71 (0.4753 to 1.075)	0.081
**Recessive model**					
PI3K –(CC + CT)	98	88	**1 (ref.)**	**1 (ref.)**	
PI3K –TT	4	25	6.96 (2.3307 to 20.785)	3.81 (1.5222 to 9.585)	0.0005
**Allele**					
PI3K –C	115	118	**1 (ref.)**	**1 (ref.)**	
PI3K –T	89	108	1.18 (0.8084 to 1.730)	1.09 (0.8932 to 1.336)	0.387
**Over dominant model**					
PI3K –CT	81	58	**1 (ref.)**	**1 (ref.)**	
PI3K CC + TT	21	55	3.65 (1.9967 to 6.700)	2.10 (1.4277 to 3.115)	0.0001

**Table 6:** Association of KLF 14 rs972283 G > A genotypes with breast cancer risk.

Genotypes	Healthy Controls	Breast Cancer	OR (95% CI)	Risk Ratio (RR)	*p*-Value
	***n* = 115**	***n* = 115**			
**Codominant model**					
KLF14-GG	47	31	**1 (reference)**	**1 (reference)**	
KLF14-GA	52	45	1.31 (0.7171 to 2.4004)	1.12 (0.8681 to 1.4554)	0.37
KLF14-AA	16	39	3.69 (1.7672 to 7.7282)	2.07 (1.3204 to 3.2493)	0.0005
**Dominant model**					
KLF14-GG	47	31	**1 (reference)**	**1 (reference)**	
KLF14-(GA + AA)	68	84	1.87 (1.0753 to 3.2620)	1.34 (1.0465 to 1.7336)	0.026
**Recessive model**					
KLF14(GG + GA)	99	76	**1 (reference)**	**1 (reference)**	
KLF14-AA	16	39	3.17 (1.6507 to 6.1076)	1.94 (1.2618 to 2.9971)	0.0005
Allele					
KLF14-G	146	107	**1 (reference)**	**1 (reference)**	
KLF14-A	84	123	1.99 (1.3759 to 2.9015)	1.42 (1.1693 to 1.7295)	0.0003
**Over dominant model**					
KLF14-GA	52	45	**1 (reference)**	**1 (reference)**	
KLF14-AA/GG	63	70	1.28 (0.7599 to 2.1694)	1.13 (0.8747 to 1.4643)	0.350

**Table 7:** Association of MDM4 rs11801299 G > A genotypes with breast cancer risk.

Genotypes	Healthy Controls	Breast Cancer	OR (95% CI)	Risk Ratio (RR)	*p*-Value
	(*n* = 115)	(*n* = 115)			
**Codominant model**					
MDM4-GG	90	72	**1 (reference)**	**1 (reference)**	
MDM4-GA	24	40	2.08 (1.1509 to 3.771)	1.48 (1.0492 to 2.0918)	0.015
MDM4-AA	1	03	3.07 (0.3819 to 36.8217)	2.22 (0.4048 to 12.2007)	0.35
**Dominant model**					
MDM4-GG	90	72	**1 (reference)**	**1 (reference)**	
MDM4-(GA + AA)	25	43	2.15 (1.2010 to 3.8487)	1.51 (1.0747 to 2.1247)	0.010
**Recessive model**					
MDM4 (GG + GA)	25	43	**1 (reference)**	**1 (reference)**	
MDM4-AA	90	72	0.46 (0.2598 to 0.8326)	0.64 (0.4561 to 0.9060)	0.010
Allele					
MDM4-G	115	115	**1 (reference)**	**1 (reference)**	
MDM4-A	26	46	1.76 (1.0249 to 3.0542)	1.38 (0.9921 to 1.9324)	0.040
**Over dominant model**					
MDM4-GA	24	40	**1 (reference)**	**1 (reference)**	
MDM4-AA/GG	91	75	0.49 (0.2738 to 0.8932)	0.68 (0.4844 to 0.9660)	0.019

**Table 8:** Risk of breast cases with respect miRNAs 27a rs895819 A > G genotypes.

Genotype	Healthy Control (*n*)	Breast Cancer (*n*)	OR (95% CI)	Risk Ratio (RR)	*p*-Value
**Codominant model**					
miR27a-AA	75	65	**1 (reference)**	**1 (reference)**	
miR27a-GA	48	33	0.79 (0.4559 to 1.3803)	0.90 (0.7129 to 1.1463)	0.41
miR27a-GG	07	17	2.80 (1.0937 to 7.1793)	1.83 (0.9663 to 3.4913)	0.031
**Dominant model**					
miR27a-AA	75	65	**1 (reference)**	**1 (reference)**	
miR27a-(GA + GG)	55	50	1.26 (0.7645 to 2.1072)	1.12 (0.8735 to 1.4489)	0.35
**Recessive model**					
miR27a (AA + GA)	123	98	**1 (reference)**	**1 (reference)**	
miR27a-GG	07	17	3.04 (1.2155 to 7.6436)	1.90 (1.0117 to 3.5990)	0.017
**Allele**					
miR27a-A	198	163	**1 (reference)**	**1 (reference)**	
miR27a-G	62	67	1.31 (0.8774 to 1.9638)	1.15 (0.9344 to 1.4351)	0.18
**Over dominant model**					
miR27a-AG	48	33	**1 (reference)**	**1 (reference)**	
miR27a-AA/GG	82	82	1.45 (0.8488 to 2.4926)	1.18 (0.9354 to 1.5017)	0.170

**Table 9:** Estimating the correlation between the miR-196aC > T genotypes and risk of breast cancer susceptibility.

Genotypes	Controls (*n*)	Breast Cases (*n*)	OR (95% CI)	Risk Ratio (RR)	*p*-Value
**Codominant model**					
miRNA-196a–CC	100	70	**1 (ref.)**	**1(ref.)**	
miRNA-196a–CT	30	44	2.02 (1.2021 to 3.651)	1.45 (1.0714 to 1.965)	0.009
miRNA-196a–TT	05	01	0.28 (0.0327 to 2.49)	0.70 (0.4831 to 1.03)	0.25
**Dominant model**					
miRNA-196a–CC	100	70	**1 (ref.)**	**1 (ref.)**	
miRNA-196a-(CT + TT)	35	45	1.83 (1.0735 to 3.142)	1.34 (1.0177 to 1.776)	0.026
**Recessive model**					
miRNA-196a-(CC + CT)	130	114	**1 (ref.)**	**1(ref.)**	
miRNA-196a–TT	05	01	0.22 (0.0263 to 1.981)	0.63 (0.4387 to 0.931)	0.18
**Allele**					
miRNA-196a–C	230	184	**1 (ref.)**	**1 (ref.)**	
miRNA-196a–T	40	46	1.43 (0.9021 to 2.290)	1.19 (0.937 to 1.522)	0.126

**Table 10:** Association of clinicopathological characteristics of BC patients and the genotype of KLF-14 (rs972283 A > G).

Parameters	*n* = 100	%	GG	GA	AA	x^2^	df	*p* Value
Age			**26**	**40**	**34**			
Age > 40 year	65	65%	18	26	21	0.13	2	0.83
Age ≤ 40 year	35	35%	8	14	13			
stage status								
Early (I and II)	30	30%	14	07	09	10.22	2	0.006
Advanced (III and IV)	70	70%	12	33	25			
Histopathological grades								
Grade I	10	10%	4	3	3	0.19	2	0.90
Grade II	30	30%	10	09	11			
Grade								
Grade I	10	10%	4	3	3	2.07	2	0.355
Grade III	60	60%	12	28	20			
ER status								
+VE	60	60%	09	24	27	12.32	2	0.002
-VE	40	40%	17	16	07			
PR status								
+VE	70	70%	21	31	18	7.22	2	0.027
-VE	30	30%	05	09	16			
Her2-neu status								
+VE	43	43%	17	19	07	12.61	2	0.0081
-VE	57	57%	09	21	27			
Distant metastasis status								
Positive	75	75%	21	31	23	1.58	2	0.45
Negative	25	25%	05	09	11			
Herceptin treatment								
Herceptin	32	32%	09	07	16	7.49	2	0.023
No Herceptin	68	68%	17	33	18			
Tamoxifen treatment								
Tamoxifen	62	62%	16	32	14	11.76	2	0.002
No Tamoxifen	38	38%	10	08	20			

**Table 11:** MDM4 rs11801299 G > A genotypes’ association with the clinicopathological characteristics of breast cancer patients. *p* values in bold are significant.

Parameters	*n* = 100		GG	GA	AA	x^2^	df	*p* Value
Association with age group			**62**	**35**	**03**			
Age >40 year	65	65%	40	24	1	1.52	2	0.46
Age ≤ 40 year	35	35%	22	11	2			
Association with stage status								
Early (I and II)	30	30%	33	05	02	15.5	2	0.0005
Advanced (III and IV)	70	70%	29	30	1			
Association with histopathological grade								
Grade I	10	10%	5	3	2	6.51	2	0.0.038
Grade II	30	30%	16	14	0			
Association of Grade I								
Grade I	10	10%	5	3	2	6.51	2	0.38
Grade III	60	60%	41	18	1			
Association with estrogen receptor status								
Positive	60	60%	40	19	01	1.89	2	0.388
Negative	40	40%	22	16	02			
Association with progesterone receptor status								
Positive	70	70%	46	22	02	1.40	2	0.499
Negative	30	30%	16	13	01			
Association with Her2/neu status								
Positive	43	43%	34	09	01	9.45	2	**0.008**
Negative	57	57%	28	27	02			
Association with distant metastasis status								
Positive	75	75%	50	23	02	6.42	2	**0.040**
Negative	25	25%	12	17	01			
Association with herceptin treatment								
Herceptin	32	32%	50	23	02	6.65	2	**0.031**
No Herceptin	68	68%	12	17	01			
Association with tamoxifen treatment								
Tamoxifen	62	62%	44	16	02	6.08	2	0.47
No tamoxifen	38	38%	18	19	01			

**Table 12:** Association of clinicopathological characteristics of breast cancer patients and the miRNAs 27a rs895819 A > G genotype. *p* values in bold are significant.

Parameters	*n* = 100		GG	GA	AA	x^2^	df	*p* Value
**Age of cases**			60	28	12			
Age >40 year	65	65%	47	10	08	15.26	2	**0.0005**
Age ≤ 40 year	35	35%	13	18	04			
**Stage status**								
Early (I and II)	30	30%	10	16	04	14.97	2	0.0006
Advanced (III and IV)	70	70%	50	12	08			
**Histopathological grade**								
Grade I	10	10%	05	02	03	0.27	2	0.87
Grade II	30	30%	15	08	07			
**Grades**								
Grade I	10	10%	05	02	03	9.2	2	**0.010**
Grade III	60	60%	40	18	02			
**ER status**								
Positive	60	60%	43	10	07	10.03	2	**0.0058**
Negative	40	40%	17	18	05			
**PR status**								
+Ve	70	70%	44	19	07	1.16	2	0.55
-Ve	30	30%	16	09	05			
**Her2-neu status**								
+Ve	43	43%	21	17	05	5.16	2	**0.07**
-Ve	57	57%	39	11	07			
**Distant metastasis status**								
+Ve	75	75%	50	19	06	6.98	2	**0.030**
-Ve	25	25%	10	09	06			
**Herceptin treatment**								
Herceptin	32	32%	13	11	08	10.25	2	**0.0059**
No herceptin	68	68%	47	17	04			
**Tamoxifen treatment**								
Tamoxifen	62	62%	40	18	04	4.8	2	0.090
No tamoxifen	38	38%	20	10	08			

**Table 13:** Association of miR-196a C > T genotypes with clinicopathological features of the breast cancer patients. *p* values in bold are significant.

Parameters	*n* = 100	100%	CC	CT	TT	x^2^	df	*p* Value
Association with age			59	40	01			
Age >40 year	65	65%	30	28	1	4.32	2	0.11
Age ≤ 40 year	35	35%	29	12	0			
Association with stage status								
Early (I and II)	30	30%	11	19	0	9.88	2	**0.0072**
Advanced (III and IV)	70	70%	48	21	1			
Association with histopathological grade								
Grade I	10	10%	6	4	0	0.58	2	0.78
Grade II	30	30%	10	12	0			
Association of I with grade								
Grade I	10	10%	6	4	0	0.19	2	0.909
Grade III	60	60%	43	34	1			
Association with estrogen receptor status								
Positive	60	60%	37	23	0	1.78	2	0.41
Negative	40	40%	22	17	1			
Association with progesterone receptor status								
Positive	70	70%	42	28	0	2.37	2	0.305
Negative	30	30%	17	12	1			
Association with Her2/neu status								
Positive	43	43%	33	10	0	10.07	2	**0.0065**
Negative	57	57%	26	30	1			
Association with distant metastasis status								
Positive	75	75%	50	24	1	8.12	2	**0.017**
Negative	25	25%	09	16	0			
Association with herceptin treatment								
Herceptin	32	32%	14	17	01	6.01	2	**0.049**
No herceptin	68	68%	45	23	0			
Association with tamoxifen treatment								
Tamoxifen	62	62%	39	22	1	1.87	2	0.39
No tamoxifen	38	38%	20	18	0			

## Data Availability

We have included the data associated with the study in the manuscript. In case of specific queries, the corresponding authors can be contacted.

## Supplementary Materials

The following supporting information can be downloaded at: https://www.mdpi.com/article/10.3390/cancers15041281/s1, Table S1: AKT1 GENE VARIENTS BT WES; Table S2: BRCA2 GENE VARIENTS OBTAINED FROM WES; Table S3: KLF14 gene varients; Figure S1: AKT-1 rs1130233 G > A gen otyping by ARMS-PCR of in Breast cancer patients; Figure S2: Phosphatidylinositol 3-kinase(PI3K)rs121913281 C > T genotyping by ARMS-PCR; Figure S3: MDM4 rs11801299 A > G genotvping by ARMS-PCR of in Breast cancer patients; Figure S4: MiR-27a rs895819 A > G genotyping by ARMS-PCR of in Breast cancer patients; Figure S5: MiR-196a2 rs11614913 C > T genotyping by ARMS-PCR of in Breast cancer patients.
