# Supplementary material for: Potential Impact of PI3K-AKT Signaling Pathway Genes, KLF-14, MDM4, miRNAs 27a, miRNA-196a Genetic Alterations in the Predisposition and Progression of Breast Cancer Patients
Source: Cancers (Basel). 2023 Feb 17;15(4):1281. doi: 10.3390/cancers15041281 (PMC9954638; doi:10.3390/cancers15041281)
Supplement: Supplementary file 1 [file cancers-15-01281-s001.zip › Figure S1.pdf]

Figure S1 AKT-1 rs1130233 G>A genotyping by ARMS -PCR of in Breast cancer patients

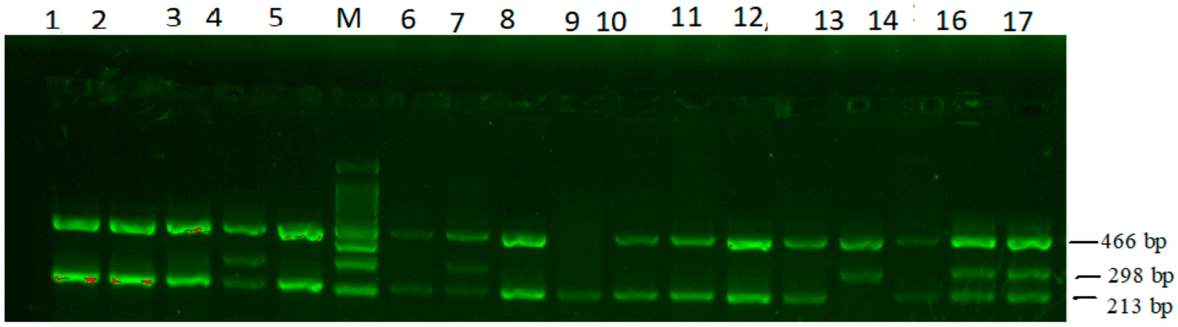

Figure S1
Legend
M-100 bp DNA ladder
Heterozygous A>G genotype: P4, P7, P16, P17
Heterozygous AA (298 bp)-P14
Homozygous GG genotype (213 bp) -P1, P2, P3, P5, P8, to P13, P15
