# Supplementary material for: Potential Impact of PI3K-AKT Signaling Pathway Genes, KLF-14, MDM4, miRNAs 27a, miRNA-196a Genetic Alterations in the Predisposition and Progression of Breast Cancer Patients
Source: Cancers (Basel). 2023 Feb 17;15(4):1281. doi: 10.3390/cancers15041281 (PMC9954638; doi:10.3390/cancers15041281)
Supplement: Supplementary file 1 [file cancers-15-01281-s001.zip › Figure S2.pdf]

FIGURE S2 Phosphatidylinositol 3-kinase (PI3K) rs121913281 C>T genotyping by ARMS-PCR.

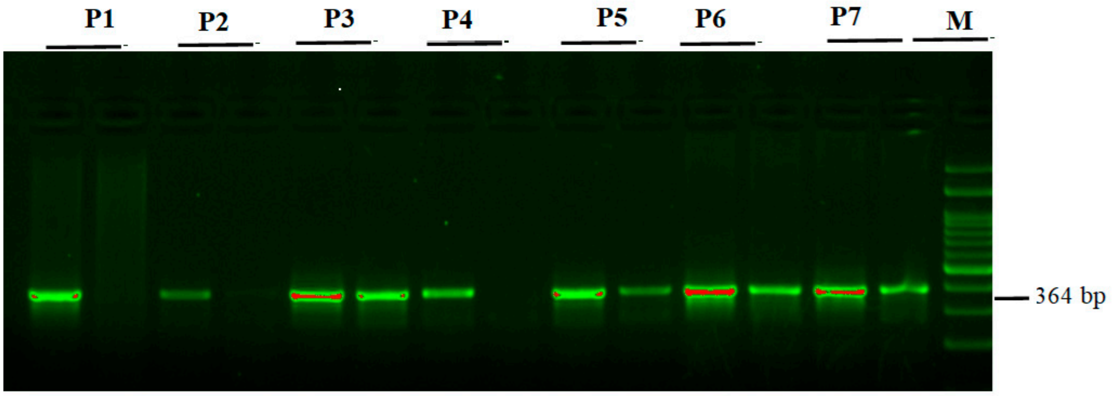

Legend

M-100 bp DNA ladder
Heterozygous CT genotype: P3,P5, P6,P7
Heterozygous TT (364 bp)-0
Homozygous CC genotype (364 bp) -P1,P2,P4
