# Supplementary material for: Potential Impact of PI3K-AKT Signaling Pathway Genes, KLF-14, MDM4, miRNAs 27a, miRNA-196a Genetic Alterations in the Predisposition and Progression of Breast Cancer Patients
Source: Cancers (Basel). 2023 Feb 17;15(4):1281. doi: 10.3390/cancers15041281 (PMC9954638; doi:10.3390/cancers15041281)
Supplement: Supplementary file 1 [file cancers-15-01281-s001.zip › Figure S3.pdf]

FIGURE S3 *MDM4* rs11801299 A>G genotyping by ARMS -PCR of in Breast cancer patients

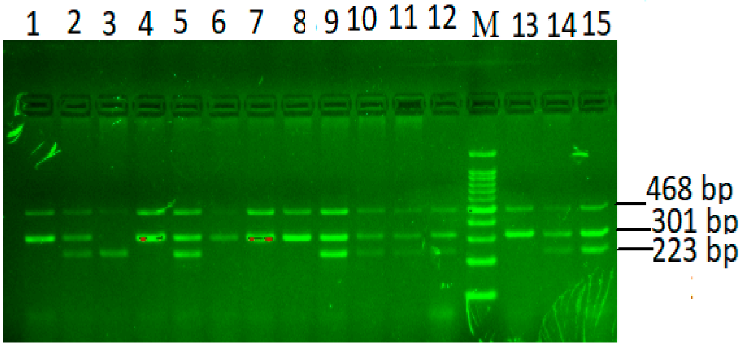

Legend

M-100 bp DNA ladder

Heterozygous A>G-P2,P5,P9,P10,P11,p12,P14,P15

Homozygous GG genotype-P1,P4,P6,P7,P8,P13

Homozygous AA genotype-P3
