# Supplementary material for: Potential Impact of PI3K-AKT Signaling Pathway Genes, KLF-14, MDM4, miRNAs 27a, miRNA-196a Genetic Alterations in the Predisposition and Progression of Breast Cancer Patients
Source: Cancers (Basel). 2023 Feb 17;15(4):1281. doi: 10.3390/cancers15041281 (PMC9954638; doi:10.3390/cancers15041281)
Supplement: Supplementary file 1 [file cancers-15-01281-s001.zip › Figure S4.pdf]

FIGURE S4 *MiR-27a rs895819 A>G* genotyping by ARMS -PCR of in Breast cancer patients.

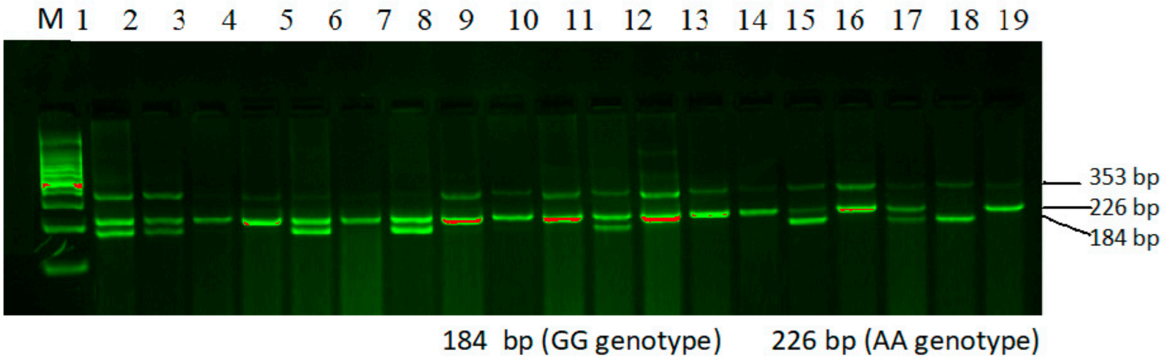

Legend

M-100 bp DNA ladder
Heterozygous A>G-P1,P2,P5,P7,P11,P15,P17
Homozygous AA genotype-P3,P4,P6,P8,P9,P10,P12,P13,P14,P15,P17,P19
Homozygous GG genotype-P18
