# Supplementary material for: Potential Impact of PI3K-AKT Signaling Pathway Genes, KLF-14, MDM4, miRNAs 27a, miRNA-196a Genetic Alterations in the Predisposition and Progression of Breast Cancer Patients
Source: Cancers (Basel). 2023 Feb 17;15(4):1281. doi: 10.3390/cancers15041281 (PMC9954638; doi:10.3390/cancers15041281)
Supplement: Supplementary file 1 [file cancers-15-01281-s001.zip › Figure S5.pdf]

Figure S5 MiR-196a2 rs11614913 C>T genotyping by ARMS -PCR of in Breast cancer patients

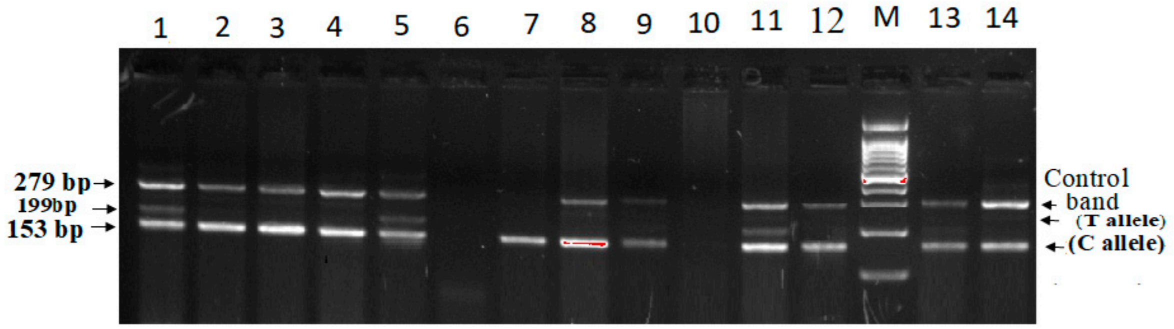

Figure S5

Legend

M-100 bp DNA ladder

Heterozygous C/T-P1, P5, P11

Homozygous CC genotype-P2,P3,P4,P7,P8,P9,P12,P13,P14

Homozygous TT genotype-0

Figure 7: MiR-196a2 rs11614913 C>T genotyping by ARMS -PCR of in Breast cancer patients.
