# Supplementary material for: Potential Impact of PI3K-AKT Signaling Pathway Genes, KLF-14, MDM4, miRNAs 27a, miRNA-196a Genetic Alterations in the Predisposition and Progression of Breast Cancer Patients
Source: Cancers (Basel). 2023 Feb 17;15(4):1281. doi: 10.3390/cancers15041281 (PMC9954638; doi:10.3390/cancers15041281)
Supplement: Supplementary file 1 [file cancers-15-01281-s001.zip › Suple Table S1.pdf]

	AKT1 GENE VARIENTS BT WES													
Chromosome	Start_Position	End_Position	Strand	Hugo_Symbol	dbSNP_RS	Reference_Alle	Tumor_Seq	Variant_Cla	Variant_Type	Consequence	VARIANT_CLAS	Tumor_Sample_Barcod	HGVSc	HGVSp
chr14	104773557	104773557	+	AKT1	rs1130233	C	T	Silent	SNP	synonymous_variant	SNV	B15_S10.vcf	c.726G>A	p.Glu242=
chr14	104773557	104773557	+	AKT1	rs1130233	C	T	Silent	SNP	synonymous_variant	SNV	B38_S18.vcf	c.726G>A	p.Glu242=
chr14	104773557	104773557	+	AKT1	rs1130233	C	T	Silent	SNP	synonymous_variant	SNV	B68_S14.vcf	c.726G>A	p.Glu242=
chr14	104773557	104773557	+	AKT1	rs1130233	C	T	Silent	SNP	synonymous_variant	SNV	B74_S4.vcf	c.726G>A	p.Glu242=
chr14	104773557	104773557	+	AKT1	rs1130233	C	T	Silent	SNP	synonymous_variant	SNV	B75_S2.vcf	c.726G>A	p.Glu242=
chr14	104774967	104774967	+	AKT1	rs2230506	G	A	Silent	SNP	synonymous_variant	SNV	B66_S15.vcf	c.604C>T	p.Leu202=
chr14	104773557	104773557	+	AKT1	rs1130233	C	T	Silent	SNP	synonymous_variant	SNV	B15_S10.vcf	c.726G>A	p.Glu242=
chr14	104773557	104773557	+	AKT1	rs1130233	C	T	Silent	SNP	synonymous_variant	SNV	B38_S18.vcf	c.726G>A	p.Glu242=
chr14	104773557	104773557	+	AKT1	rs1130233	C	T	Silent	SNP	synonymous_variant	SNV	B68_S14.vcf	c.726G>A	p.Glu242=
chr14	104773557	104773557	+	AKT1	rs1130233	C	T	Silent	SNP	synonymous_variant	SNV	B74_S4.vcf	c.726G>A	p.Glu242=
chr14	104773557	104773557	+	AKT1	rs1130233	C	T	Silent	SNP	synonymous_variant	SNV	B75_S2.vcf	c.726G>A	p.Glu242=

HGVSp_Sho	Exon_Numb	Freq_in_21	Percentage
p.E242=	8/13	5	23.809524
p.E242=	8/13	5	23.809524
p.E242=	8/13	5	23.809524
p.E242=	8/13	5	23.809524
p.E242=	8/13	5	23.809524
p.L202=	6/13	1	4.7619048
p.E242=	8/13	5	23.809524
p.E242=	8/13	5	23.809524
p.E242=	8/13	5	23.809524
p.E242=	8/13	5	23.809524
p.E242=	8/13	5	23.809524
