# Supplementary material for: Potential Impact of PI3K-AKT Signaling Pathway Genes, KLF-14, MDM4, miRNAs 27a, miRNA-196a Genetic Alterations in the Predisposition and Progression of Breast Cancer Patients
Source: Cancers (Basel). 2023 Feb 17;15(4):1281. doi: 10.3390/cancers15041281 (PMC9954638; doi:10.3390/cancers15041281)
Supplement: Supplementary file 1 [file cancers-15-01281-s001.zip › Supple Table S2.pdf]

BRCA2 GENE VARIENTS OBTAINED FROM WES

Chromosome	Start_Position	End_Position	Strand	Hugo_Symbo	dbSNP_RS	eference_Alle	mor_Seq_Alle
chr17	43092919	43092919	+	BRCA1	rs799917	G	A
chr17	43092919	43092919	+	BRCA1	rs799917	G	A
chr17	43092919	43092919	+	BRCA1	rs799917	G	A
chr17	43092919	43092919	+	BRCA1	rs799917	G	A
chr17	43092919	43092919	+	BRCA1	rs799917	G	A
chr17	43092919	43092919	+	BRCA1	rs799917	G	A
chr17	43092919	43092919	+	BRCA1	rs799917	G	A
chr17	43092919	43092919	+	BRCA1	rs799917	G	A
chr17	43092919	43092919	+	BRCA1	rs799917	G	A
chr17	43092919	43092919	+	BRCA1	rs799917	G	A
chr17	43092919	43092919	+	BRCA1	rs799917	G	A
chr17	43092919	43092919	+	BRCA1	rs799917	G	A
chr17	43092919	43092919	+	BRCA1	rs799917	G	A
chr17	43092919	43092919	+	BRCA1	rs799917	G	A
chr17	43071077	43071077	+	BRCA1	rs1799966	T	C
chr17	43091983	43091983	+	BRCA1	rs16942	T	C
chr17	43093449	43093449	+	BRCA1	rs1799949	G	A
chr17	43071077	43071077	+	BRCA1	rs1799966	T	C
chr17	43091983	43091983	+	BRCA1	rs16942	T	C
chr17	43093449	43093449	+	BRCA1	rs1799949	G	A
chr17	43071077	43071077	+	BRCA1	rs1799966	T	C
chr17	43091983	43091983	+	BRCA1	rs16942	T	C
chr17	43093449	43093449	+	BRCA1	rs1799949	G	A
chr17	43071077	43071077	+	BRCA1	rs1799966	T	C
chr17	43091983	43091983	+	BRCA1	rs16942	T	C
chr17	43093449	43093449	+	BRCA1	rs1799949	G	A
chr17	43071077	43071077	+	BRCA1	rs1799966	T	C
chr17	43091983	43091983	+	BRCA1	rs16942	T	C
chr17	43093449	43093449	+	BRCA1	rs1799949	G	A
chr17	43071077	43071077	+	BRCA1	rs1799966	T	C
chr17	43091983	43091983	+	BRCA1	rs16942	T	C
chr17	43093449	43093449	+	BRCA1	rs1799949	G	A
chr17	43071077	43071077	+	BRCA1	rs1799966	T	C
chr17	43091983	43091983	+	BRCA1	rs16942	T	C
chr17	43093449	43093449	+	BRCA1	rs1799949	G	A
chr17	43071077	43071077	+	BRCA1	rs1799966	T	C
chr17	43091983	43091983	+	BRCA1	rs16942	T	C
chr17	43093449	43093449	+	BRCA1	rs1799949	G	A
chr17	43071077	43071077	+	BRCA1	rs1799966	T	C
chr17	43091983	43091983	+	BRCA1	rs16942	T	C
chr17	43093449	43093449	+	BRCA1	rs1799949	G	A
chr17	43071077	43071077	+	BRCA1	rs1799966	T	C
chr17	43091983	43091983	+	BRCA1	rs16942	T	C
chr17	43093449	43093449	+	BRCA1	rs1799949	G	A
chr17	43071077	43071077	+	BRCA1	rs1799966	T	C
chr17	43091983	43091983	+	BRCA1	rs16942	T	C
chr17	43093449	43093449	+	BRCA1	rs1799949	G	A
chr17	43071077	43071077	+	BRCA1	rs1799966	T	C
chr17	43091983	43091983	+	BRCA1	rs16942	T	C
chr17	43093449	43093449	+	BRCA1	rs1799949	G	A
chr17	43071077	43071077	+	BRCA1	rs1799966	T	C
chr17	43091983	43091983	+	BRCA1	rs16942	T	C
chr17	43093449	43093449	+	BRCA1	rs1799949	G	A
chr17	43082453	43082453	+	BRCA1	rs1060915	A	G

chr17	43092418	43092418	+	BRCA1	rs16941	T	C
chr17	43082453	43082453	+	BRCA1	rs1060915	A	G
chr17	43092418	43092418	+	BRCA1	rs16941	T	C
chr17	43082453	43082453	+	BRCA1	rs1060915	A	G
chr17	43092418	43092418	+	BRCA1	rs16941	T	C
chr17	43082453	43082453	+	BRCA1	rs1060915	A	G
chr17	43092418	43092418	+	BRCA1	rs16941	T	C
chr17	43082453	43082453	+	BRCA1	rs1060915	A	G
chr17	43092418	43092418	+	BRCA1	rs16941	T	C
chr17	43082453	43082453	+	BRCA1	rs1060915	A	G
chr17	43092418	43092418	+	BRCA1	rs16941	T	C
chr17	43082453	43082453	+	BRCA1	rs1060915	A	G
chr17	43092418	43092418	+	BRCA1	rs16941	T	C
chr17	43082453	43082453	+	BRCA1	rs1060915	A	G
chr17	43092418	43092418	+	BRCA1	rs16941	T	C
chr17	43082453	43082453	+	BRCA1	rs1060915	A	G
chr17	43092418	43092418	+	BRCA1	rs16941	T	C
chr17	43082453	43082453	+	BRCA1	rs1060915	A	G
chr17	43092418	43092418	+	BRCA1	rs16941	T	C
chr17	43082453	43082453	+	BRCA1	rs1060915	A	G
chr17	43092418	43092418	+	BRCA1	rs16941	T	C
chr17	43082453	43082453	+	BRCA1	rs1060915	A	G
chr17	43092418	43092418	+	BRCA1	rs16941	T	C
chr7	130733698	130733698	+	KLF14	rs76603546	G	A
chr17	43093220	43093220	+	BRCA1	rs16940	A	G
chr17	43093220	43093220	+	BRCA1	rs16940	A	G
chr7	130733698	130733698	+	KLF14	rs76603546	G	A
chr17	43093220	43093220	+	BRCA1	rs16940	A	G
chr7	130733698	130733698	+	KLF14	rs76603546	G	A
chr7	130733698	130733698	+	KLF14	rs76603546	G	A
chr17	43093220	43093220	+	BRCA1	rs16940	A	G
chr17	43093220	43093220	+	BRCA1	rs16940	A	G
chr7	130733698	130733698	+	KLF14	rs76603546	G	A
chr17	43093220	43093220	+	BRCA1	rs16940	A	G
chr7	130733698	130733698	+	KLF14	rs76603546	G	A
chr17	43093220	43093220	+	BRCA1	rs16940	A	G
chr7	130733698	130733698	+	KLF14	rs76603546	G	A
chr7	130733698	130733698	+	KLF14	rs76603546	G	A
chr17	43093220	43093220	+	BRCA1	rs16940	A	G
chr7	130733698	130733698	+	KLF14	rs76603546	G	A
chr17	43093220	43093220	+	BRCA1	rs16940	A	G
chr7	130733698	130733698	+	KLF14	rs76603546	G	A
chr17	43093220	43093220	+	BRCA1	rs16940	A	G
chr7	130733698	130733698	+	KLF14	rs76603546	G	A
chr17	43093220	43093220	+	BRCA1	rs16940	A	G
chr14	104773557	104773557	+	AKT1	rs1130233	C	T
chr14	104773557	104773557	+	AKT1	rs1130233	C	T
chr14	104773557	104773557	+	AKT1	rs1130233	C	T
chr14	104773557	104773557	+	AKT1	rs1130233	C	T
chr14	104773557	104773557	+	AKT1	rs1130233	C	T
chr2	241851281	241851281	+	PDCD1	rs2227982	G	A
chr2	241851281	241851281	+	PDCD1	rs2227982	G	A
chr17	43093454	43093454	+	BRCA1	rs4986850	C	T
chr17	43093454	43093454	+	BRCA1	rs4986850	C	T
chr2	241851281	241851281	+	PDCD1	rs2227982	G	A
chr17	43093454	43093454	+	BRCA1	rs4986850	C	T
chr2	241851281	241851281	+	PDCD1	rs2227982	G	A

chr17	43093454	43093454	+	BRCA1	rs4986850	C	T
chr17	43091814	43091814	+	BRCA1	rs730881453	A	T
chr17	43091814	43091814	+	BRCA1	rs730881453	A	T
chr17	43091814	43091814	+	BRCA1	rs730881453	A	T
chr17	43094464	43094464	+	BRCA1	rs1799950	T	C
chr17	43094464	43094464	+	BRCA1	rs1799950	T	C
chr17	43115746	43115746	+	BRCA1	rs1800062	C	T

ant_Classifica	Variant_Type	Consequence	ARIANT_CLAS	r_Sample_Ba	HGVSc	HGVSp	HGVSp_Short
ssense_Mutat	SNP	issense_varia	SNV	B21_S6.vcf	c.2612C>T	p.Pro871Leu	p.P871L
ssense_Mutat	SNP	issense_varia	SNV	B26_S12.vcf	c.2612C>T	p.Pro871Leu	p.P871L
ssense_Mutat	SNP	issense_varia	SNV	B35_S74.vcf	c.2612C>T	p.Pro871Leu	p.P871L
ssense_Mutat	SNP	issense_varia	SNV	B40_S5.vcf	c.2612C>T	p.Pro871Leu	p.P871L
ssense_Mutat	SNP	issense_varia	SNV	B63_S3.vcf	c.2612C>T	p.Pro871Leu	p.P871L
ssense_Mutat	SNP	issense_varia	SNV	B66_S15.vcf	c.2612C>T	p.Pro871Leu	p.P871L
ssense_Mutat	SNP	issense_varia	SNV	B68_S14.vcf	c.2612C>T	p.Pro871Leu	p.P871L
ssense_Mutat	SNP	issense_varia	SNV	B69_S11.vcf	c.2612C>T	p.Pro871Leu	p.P871L
ssense_Mutat	SNP	issense_varia	SNV	B71_S17.vcf	c.2612C>T	p.Pro871Leu	p.P871L
ssense_Mutat	SNP	issense_varia	SNV	B74_S4.vcf	c.2612C>T	p.Pro871Leu	p.P871L
ssense_Mutat	SNP	issense_varia	SNV	B75_S2.vcf	c.2612C>T	p.Pro871Leu	p.P871L
ssense_Mutat	SNP	issense_varia	SNV	B76_S1.vcf	c.2612C>T	p.Pro871Leu	p.P871L
ssense_Mutat	SNP	issense_varia	SNV	B79_S7.vcf	c.2612C>T	p.Pro871Leu	p.P871L
ssense_Mutat	SNP	issense_varia	SNV	B86_S73.vcf	c.2612C>T	p.Pro871Leu	p.P871L
ssense_Mutat	SNP	issense_varia	SNV	B18_S13.vcf	c.4900A>G	p.Ser1634Gly	p.S1634G
ssense_Mutat	SNP	issense_varia	SNV	B18_S13.vcf	c.3548A>G	p.Lys1183Arg	p.K1183R
Silent	SNP	onymous_var	SNV	B18_S13.vcf	c.2082C>T	p.Ser694=	p.S694=
ssense_Mutat	SNP	issense_varia	SNV	B21_S6.vcf	c.4900A>G	p.Ser1634Gly	p.S1634G
ssense_Mutat	SNP	issense_varia	SNV	B21_S6.vcf	c.3548A>G	p.Lys1183Arg	p.K1183R
Silent	SNP	onymous_var	SNV	B21_S6.vcf	c.2082C>T	p.Ser694=	p.S694=
ssense_Mutat	SNP	issense_varia	SNV	B35_S74.vcf	c.4900A>G	p.Ser1634Gly	p.S1634G
ssense_Mutat	SNP	issense_varia	SNV	B35_S74.vcf	c.3548A>G	p.Lys1183Arg	p.K1183R
Silent	SNP	onymous_var	SNV	B35_S74.vcf	c.2082C>T	p.Ser694=	p.S694=
ssense_Mutat	SNP	issense_varia	SNV	B63_S3.vcf	c.4900A>G	p.Ser1634Gly	p.S1634G
ssense_Mutat	SNP	issense_varia	SNV	B63_S3.vcf	c.3548A>G	p.Lys1183Arg	p.K1183R
Silent	SNP	onymous_var	SNV	B63_S3.vcf	c.2082C>T	p.Ser694=	p.S694=
ssense_Mutat	SNP	issense_varia	SNV	B66_S15.vcf	c.4900A>G	p.Ser1634Gly	p.S1634G
ssense_Mutat	SNP	issense_varia	SNV	B66_S15.vcf	c.3548A>G	p.Lys1183Arg	p.K1183R
Silent	SNP	onymous_var	SNV	B66_S15.vcf	c.2082C>T	p.Ser694=	p.S694=
ssense_Mutat	SNP	issense_varia	SNV	B68_S14.vcf	c.4900A>G	p.Ser1634Gly	p.S1634G
ssense_Mutat	SNP	issense_varia	SNV	B68_S14.vcf	c.3548A>G	p.Lys1183Arg	p.K1183R
Silent	SNP	onymous_var	SNV	B68_S14.vcf	c.2082C>T	p.Ser694=	p.S694=
ssense_Mutat	SNP	issense_varia	SNV	B69_S11.vcf	c.4900A>G	p.Ser1634Gly	p.S1634G
ssense_Mutat	SNP	issense_varia	SNV	B69_S11.vcf	c.3548A>G	p.Lys1183Arg	p.K1183R
Silent	SNP	onymous_var	SNV	B69_S11.vcf	c.2082C>T	p.Ser694=	p.S694=
ssense_Mutat	SNP	issense_varia	SNV	B71_S17.vcf	c.4900A>G	p.Ser1634Gly	p.S1634G
ssense_Mutat	SNP	issense_varia	SNV	B71_S17.vcf	c.3548A>G	p.Lys1183Arg	p.K1183R
Silent	SNP	onymous_var	SNV	B71_S17.vcf	c.2082C>T	p.Ser694=	p.S694=
ssense_Mutat	SNP	issense_varia	SNV	B74_S4.vcf	c.4900A>G	p.Ser1634Gly	p.S1634G
ssense_Mutat	SNP	issense_varia	SNV	B74_S4.vcf	c.3548A>G	p.Lys1183Arg	p.K1183R
Silent	SNP	onymous_var	SNV	B74_S4.vcf	c.2082C>T	p.Ser694=	p.S694=
ssense_Mutat	SNP	issense_varia	SNV	B75_S2.vcf	c.4900A>G	p.Ser1634Gly	p.S1634G
ssense_Mutat	SNP	issense_varia	SNV	B75_S2.vcf	c.3548A>G	p.Lys1183Arg	p.K1183R
Silent	SNP	onymous_var	SNV	B75_S2.vcf	c.2082C>T	p.Ser694=	p.S694=
ssense_Mutat	SNP	issense_varia	SNV	B76_S1.vcf	c.4900A>G	p.Ser1634Gly	p.S1634G
ssense_Mutat	SNP	issense_varia	SNV	B76_S1.vcf	c.3548A>G	p.Lys1183Arg	p.K1183R
Silent	SNP	onymous_var	SNV	B76_S1.vcf	c.2082C>T	p.Ser694=	p.S694=
ssense_Mutat	SNP	issense_varia	SNV	B79_S7.vcf	c.4900A>G	p.Ser1634Gly	p.S1634G
ssense_Mutat	SNP	issense_varia	SNV	B79_S7.vcf	c.3548A>G	p.Lys1183Arg	p.K1183R
Silent	SNP	onymous_var	SNV	B79_S7.vcf	c.2082C>T	p.Ser694=	p.S694=
ssense_Mutat	SNP	issense_varia	SNV	B86_S73.vcf	c.4900A>G	p.Ser1634Gly	p.S1634G
ssense_Mutat	SNP	issense_varia	SNV	B86_S73.vcf	c.3548A>G	p.Lys1183Arg	p.K1183R
Silent	SNP	onymous_var	SNV	B86_S73.vcf	c.2082C>T	p.Ser694=	p.S694=
Silent	SNP	onymous_var	SNV	B18_S13.vcf	c.4308T>C	p.Ser1436=	p.S1436=

ssense_Mutat	SNP	issense_varia	SNV	B18_S13.vcf	c.3113A>G	p.Glu1038Gly	p.E1038G
Silent	SNP	onymous_var	SNV	B21_S6.vcf	c.4308T>C	p.Ser1436=	p.S1436=
ssense_Mutat	SNP	issense_varia	SNV	B21_S6.vcf	c.3113A>G	p.Glu1038Gly	p.E1038G
Silent	SNP	onymous_var	SNV	B35_S74.vcf	c.4308T>C	p.Ser1436=	p.S1436=
ssense_Mutat	SNP	issense_varia	SNV	B35_S74.vcf	c.3113A>G	p.Glu1038Gly	p.E1038G
Silent	SNP	onymous_var	SNV	B63_S3.vcf	c.4308T>C	p.Ser1436=	p.S1436=
ssense_Mutat	SNP	issense_varia	SNV	B63_S3.vcf	c.3113A>G	p.Glu1038Gly	p.E1038G
Silent	SNP	onymous_var	SNV	B66_S15.vcf	c.4308T>C	p.Ser1436=	p.S1436=
ssense_Mutat	SNP	issense_varia	SNV	B66_S15.vcf	c.3113A>G	p.Glu1038Gly	p.E1038G
Silent	SNP	onymous_var	SNV	B68_S14.vcf	c.4308T>C	p.Ser1436=	p.S1436=
ssense_Mutat	SNP	issense_varia	SNV	B68_S14.vcf	c.3113A>G	p.Glu1038Gly	p.E1038G
Silent	SNP	onymous_var	SNV	B69_S11.vcf	c.4308T>C	p.Ser1436=	p.S1436=
ssense_Mutat	SNP	issense_varia	SNV	B69_S11.vcf	c.3113A>G	p.Glu1038Gly	p.E1038G
Silent	SNP	onymous_var	SNV	B71_S17.vcf	c.4308T>C	p.Ser1436=	p.S1436=
ssense_Mutat	SNP	issense_varia	SNV	B71_S17.vcf	c.3113A>G	p.Glu1038Gly	p.E1038G
Silent	SNP	onymous_var	SNV	B75_S2.vcf	c.4308T>C	p.Ser1436=	p.S1436=
ssense_Mutat	SNP	issense_varia	SNV	B75_S2.vcf	c.3113A>G	p.Glu1038Gly	p.E1038G
Silent	SNP	onymous_var	SNV	B76_S1.vcf	c.4308T>C	p.Ser1436=	p.S1436=
ssense_Mutat	SNP	issense_varia	SNV	B76_S1.vcf	c.3113A>G	p.Glu1038Gly	p.E1038G
Silent	SNP	onymous_var	SNV	B79_S7.vcf	c.4308T>C	p.Ser1436=	p.S1436=
ssense_Mutat	SNP	issense_varia	SNV	B79_S7.vcf	c.3113A>G	p.Glu1038Gly	p.E1038G
Silent	SNP	onymous_var	SNV	B86_S73.vcf	c.4308T>C	p.Ser1436=	p.S1436=
ssense_Mutat	SNP	issense_varia	SNV	B86_S73.vcf	c.3113A>G	p.Glu1038Gly	p.E1038G
Silent	SNP	onymous_var	SNV	B15_S10.vcf	c.336C>T	p.Ser112=	p.S112=
Silent	SNP	onymous_var	SNV	B18_S13.vcf	c.2311T>C	p.Leu771=	p.L771=
Silent	SNP	onymous_var	SNV	B21_S6.vcf	c.2311T>C	p.Leu771=	p.L771=
Silent	SNP	onymous_var	SNV	B35_S74.vcf	c.336C>T	p.Ser112=	p.S112=
Silent	SNP	onymous_var	SNV	B35_S74.vcf	c.2311T>C	p.Leu771=	p.L771=
Silent	SNP	onymous_var	SNV	B38_S18.vcf	c.336C>T	p.Ser112=	p.S112=
Silent	SNP	onymous_var	SNV	B40_S5.vcf	c.336C>T	p.Ser112=	p.S112=
Silent	SNP	onymous_var	SNV	B63_S3.vcf	c.2311T>C	p.Leu771=	p.L771=
Silent	SNP	onymous_var	SNV	B66_S15.vcf	c.2311T>C	p.Leu771=	p.L771=
Silent	SNP	onymous_var	SNV	B68_S14.vcf	c.336C>T	p.Ser112=	p.S112=
Silent	SNP	onymous_var	SNV	B68_S14.vcf	c.2311T>C	p.Leu771=	p.L771=
Silent	SNP	onymous_var	SNV	B71_S17.vcf	c.336C>T	p.Ser112=	p.S112=
Silent	SNP	onymous_var	SNV	B71_S17.vcf	c.2311T>C	p.Leu771=	p.L771=
Silent	SNP	onymous_var	SNV	B74_S4.vcf	c.336C>T	p.Ser112=	p.S112=
Silent	SNP	onymous_var	SNV	B75_S2.vcf	c.336C>T	p.Ser112=	p.S112=
Silent	SNP	onymous_var	SNV	B75_S2.vcf	c.2311T>C	p.Leu771=	p.L771=
Silent	SNP	onymous_var	SNV	B76_S1.vcf	c.336C>T	p.Ser112=	p.S112=
Silent	SNP	onymous_var	SNV	B76_S1.vcf	c.2311T>C	p.Leu771=	p.L771=
Silent	SNP	onymous_var	SNV	B77_S8.vcf	c.336C>T	p.Ser112=	p.S112=
Silent	SNP	onymous_var	SNV	B79_S7.vcf	c.2311T>C	p.Leu771=	p.L771=
Silent	SNP	onymous_var	SNV	B86_S73.vcf	c.336C>T	p.Ser112=	p.S112=
Silent	SNP	onymous_var	SNV	B86_S73.vcf	c.2311T>C	p.Leu771=	p.L771=
Silent	SNP	onymous_var	SNV	B15_S10.vcf	c.726G>A	p.Glu242=	p.E242=
Silent	SNP	onymous_var	SNV	B38_S18.vcf	c.726G>A	p.Glu242=	p.E242=
Silent	SNP	onymous_var	SNV	B68_S14.vcf	c.726G>A	p.Glu242=	p.E242=
Silent	SNP	onymous_var	SNV	B74_S4.vcf	c.726G>A	p.Glu242=	p.E242=
Silent	SNP	onymous_var	SNV	B75_S2.vcf	c.726G>A	p.Glu242=	p.E242=
ssense_Mutat	SNP	issense_varia	SNV	B15_S10.vcf	c.644C>T	p.Ala215Val	p.A215V
ssense_Mutat	SNP	issense_varia	SNV	B18_S13.vcf	c.644C>T	p.Ala215Val	p.A215V
ssense_Mutat	SNP	issense_varia	SNV	B18_S13.vcf	c.2077G>A	p.Asp693Asn	p.D693N
ssense_Mutat	SNP	issense_varia	SNV	B35_S74.vcf	c.2077G>A	p.Asp693Asn	p.D693N
ssense_Mutat	SNP	issense_varia	SNV	B69_S11.vcf	c.644C>T	p.Ala215Val	p.A215V
ssense_Mutat	SNP	issense_varia	SNV	B69_S11.vcf	c.2077G>A	p.Asp693Asn	p.D693N
ssense_Mutat	SNP	issense_varia	SNV	B79_S7.vcf	c.644C>T	p.Ala215Val	p.A215V

ssense_Mutat	SNP	issense_varia	SNV	B79_S7.vcf	c.2077G>A	p.Asp693Asn	p.D693N
Silent	SNP	onymous_var	SNV	B18_S13.vcf	c.3717T>A	p.Ser1239=	p.S1239=
Silent	SNP	onymous_var	SNV	B69_S11.vcf	c.3717T>A	p.Ser1239=	p.S1239=
Silent	SNP	onymous_var	SNV	B79_S7.vcf	c.3717T>A	p.Ser1239=	p.S1239=
ssense_Mutat	SNP	issense_varia	SNV	B76_S1.vcf	c.1067A>G	p.Gln356Arg	p.Q356R
ssense_Mutat	SNP	issense_varia	SNV	B86_S73.vcf	c.1067A>G	p.Gln356Arg	p.Q356R
Silent	SNP	onymous_var	SNV	B15_S10.vcf	c.114G>A	p.Lys38=	p.K38=

Exon_Number	Freq_in_21	Percentage
10/24	15	71.4285714
10/24	15	71.4285714
10/24	15	71.4285714
10/24	15	71.4285714
10/24	15	71.4285714
10/24	15	71.4285714
10/24	15	71.4285714
10/24	15	71.4285714
10/24	15	71.4285714
10/24	15	71.4285714
10/24	15	71.4285714
10/24	15	71.4285714
10/24	15	71.4285714
10/24	15	71.4285714
16/24	13	61.9047619
10/24	13	61.9047619
10/24	13	61.9047619
16/24	13	61.9047619
10/24	13	61.9047619
10/24	13	61.9047619
16/24	13	61.9047619
10/24	13	61.9047619
10/24	13	61.9047619
16/24	13	61.9047619
10/24	13	61.9047619
10/24	13	61.9047619
16/24	13	61.9047619
10/24	13	61.9047619
10/24	13	61.9047619
16/24	13	61.9047619
10/24	13	61.9047619
10/24	13	61.9047619
16/24	13	61.9047619
10/24	13	61.9047619
10/24	13	61.9047619
16/24	13	61.9047619
10/24	13	61.9047619
10/24	13	61.9047619
16/24	13	61.9047619
10/24	13	61.9047619
10/24	13	61.9047619
16/24	13	61.9047619
10/24	13	61.9047619
10/24	13	61.9047619
16/24	13	61.9047619
10/24	13	61.9047619
10/24	13	61.9047619
16/24	13	61.9047619
10/24	13	61.9047619
10/24	13	61.9047619
16/24	13	61.9047619
10/24	13	61.9047619
10/24	13	61.9047619
12/24	12	57.1428571

10/24	12	57.1428571
12/24	12	57.1428571
10/24	12	57.1428571
12/24	12	57.1428571
10/24	12	57.1428571
12/24	12	57.1428571
10/24	12	57.1428571
12/24	12	57.1428571
10/24	12	57.1428571
12/24	12	57.1428571
10/24	12	57.1428571
12/24	12	57.1428571
10/24	12	57.1428571
12/24	12	57.1428571
10/24	12	57.1428571
12/24	12	57.1428571
10/24	12	57.1428571
12/24	12	57.1428571
10/24	12	57.1428571
12/24	12	57.1428571
10/24	12	57.1428571
12/24	12	57.1428571
10/24	12	57.1428571
1/1	11	52.3809524
10/24	11	52.3809524
10/24	11	52.3809524
1/1	11	52.3809524
10/24	11	52.3809524
1/1	11	52.3809524
1/1	11	52.3809524
10/24	11	52.3809524
10/24	11	52.3809524
1/1	11	52.3809524
10/24	11	52.3809524
1/1	11	52.3809524
10/24	11	52.3809524
1/1	11	52.3809524
1/1	11	52.3809524
10/24	11	52.3809524
1/1	11	52.3809524
10/24	11	52.3809524
1/1	11	52.3809524
10/24	11	52.3809524
1/1	11	52.3809524
10/24	11	52.3809524
8/13	5	23.8095238
8/13	5	23.8095238
8/13	5	23.8095238
8/13	5	23.8095238
8/13	5	23.8095238
5/5	4	19.047619
5/5	4	19.047619
10/24	4	19.047619
10/24	4	19.047619
5/5	4	19.047619
10/24	4	19.047619
5/5	4	19.047619

10/24	4	19.047619
10/24	3	14.2857143
10/24	3	14.2857143
10/24	3	14.2857143
10/24	2	9.52380952
10/24	2	9.52380952
3/24	1	4.76190476
