# Supplementary material for: Potential Impact of PI3K-AKT Signaling Pathway Genes, KLF-14, MDM4, miRNAs 27a, miRNA-196a Genetic Alterations in the Predisposition and Progression of Breast Cancer Patients
Source: Cancers (Basel). 2023 Feb 17;15(4):1281. doi: 10.3390/cancers15041281 (PMC9954638; doi:10.3390/cancers15041281)
Supplement: Supplementary file 1 [file cancers-15-01281-s001.zip › Supple table S3.pdf]

	KLF14 gene varients						
Chromosome	Start_Position	End_Position	Strand	Hugo_Symbol	dbSNP_RS	eference_Alle	mor_Seq_Alle
chr7	130733862	130733862	+	KLF14	rs111400400	G	A
chr7	130733894	130733894	+	KLF14	rs184537657	G	T
chr7	130733917	130733917	+	KLF14	rs111731678	A	T
chr7	130733862	130733862	+	KLF14	rs111400400	G	A
chr7	130733894	130733894	+	KLF14	rs184537657	G	T
chr7	130733917	130733917	+	KLF14	rs111731678	A	T
chr7	130733862	130733862	+	KLF14	rs111400400	G	A
chr7	130733894	130733894	+	KLF14	rs184537657	G	T
chr7	130733917	130733917	+	KLF14	rs111731678	A	T
chr7	130733862	130733862	+	KLF14	rs111400400	G	A
chr7	130733894	130733894	+	KLF14	rs184537657	G	T
chr7	130733917	130733917	+	KLF14	rs111731678	A	T
chr7	130733862	130733862	+	KLF14	rs111400400	G	A
chr7	130733894	130733894	+	KLF14	rs184537657	G	T
chr7	130733917	130733917	+	KLF14	rs111731678	A	T
chr7	130733862	130733862	+	KLF14	rs111400400	G	A
chr7	130733894	130733894	+	KLF14	rs184537657	G	T
chr7	130733917	130733917	+	KLF14	rs111731678	A	T
chr7	130733862	130733862	+	KLF14	rs111400400	G	A
chr7	130733894	130733894	+	KLF14	rs184537657	G	T
chr7	130733917	130733917	+	KLF14	rs111731678	A	T
chr7	130733862	130733862	+	KLF14	rs111400400	G	A
chr7	130733894	130733894	+	KLF14	rs184537657	G	T
chr7	130733917	130733917	+	KLF14	rs111731678	A	T
chr7	130733862	130733862	+	KLF14	rs111400400	G	A
chr7	130733894	130733894	+	KLF14	rs184537657	G	T
chr7	130733917	130733917	+	KLF14	rs111731678	A	T
chr7	130733862	130733862	+	KLF14	rs111400400	G	A
chr7	130733894	130733894	+	KLF14	rs184537657	G	T
chr7	130733917	130733917	+	KLF14	rs111731678	A	T
chr7	130733862	130733862	+	KLF14	rs111400400	G	A
chr7	130733894	130733894	+	KLF14	rs184537657	G	T
chr7	130733917	130733917	+	KLF14	rs111731678	A	T
chr7	130733862	130733862	+	KLF14	rs111400400	G	A
chr7	130733894	130733894	+	KLF14	rs184537657	G	T
chr7	130733917	130733917	+	KLF14	rs111731678	A	T
chr7	130733862	130733862	+	KLF14	rs111400400	G	A
chr7	130733894	130733894	+	KLF14	rs184537657	G	T
chr7	130733917	130733917	+	KLF14	rs111731678	A	T
chr7	130733862	130733862	+	KLF14	rs111400400	G	A
chr7	130733894	130733894	+	KLF14	rs184537657	G	T
chr7	130733917	130733917	+	KLF14	rs111731678	A	T
chr7	130733862	130733862	+	KLF14	rs111400400	G	A
chr7	130733894	130733894	+	KLF14	rs184537657	G	T
chr7	130733917	130733917	+	KLF14	rs111731678	A	T
chr7	130733862	130733862	+	KLF14	rs111400400	G	A
chr7	130733894	130733894	+	KLF14	rs184537657	G	T
chr7	130733917	130733917	+	KLF14	rs111731678	A	T
chr7	130733862	130733862	+	KLF14	rs111400400	G	A
chr7	130733894	130733894	+	KLF14	rs184537657	G	T
chr7	130733917	130733917	+	KLF14	rs111731678	A	T
chr7	130733862	130733862	+	KLF14	rs111400400	G	A
chr7	130733894	130733894	+	KLF14	rs184537657	G	T
chr7	130733917	130733917	+	KLF14	rs111731678	A	T
chr7	130733862	130733862	+	KLF14	rs111400400	G	A

chr7	130733894	130733894	+	KLF14	rs184537657	G	T
chr7	130733917	130733917	+	KLF14	rs111731678	A	T
chr7	130733862	130733862	+	KLF14	rs111400400	G	A
chr7	130733894	130733894	+	KLF14	rs184537657	G	T
chr7	130733917	130733917	+	KLF14	rs111731678	A	T
chr7	130733862	130733862	+	KLF14	rs111400400	G	A
chr7	130733894	130733894	+	KLF14	rs184537657	G	T
chr7	130733917	130733917	+	KLF14	rs111731678	A	T
chr7	130733698	130733698	+	KLF14	rs76603546	G	A
chr7	130733698	130733698	+	KLF14	rs76603546	G	A

ant_Classifica	Variant_Type	Consequence	ARIANT_CLAS	r_Sample_Ba	HGVSc	HGVSp	HGVSp_Short
ssense_Mutat	SNP	issense_varia	SNV	B12_S9.vcf	c.172C>T	p.Pro58Ser	p.P58S
ssense_Mutat	SNP	issense_varia	SNV	B12_S9.vcf	c.140C>A	p.Pro47Gln	p.P47Q
Silent	SNP	onymous_var	SNV	B12_S9.vcf	c.117T>A	p.Ala39=	p.A39=
ssense_Mutat	SNP	issense_varia	SNV	B15_S10.vcf	c.172C>T	p.Pro58Ser	p.P58S
ssense_Mutat	SNP	issense_varia	SNV	B15_S10.vcf	c.140C>A	p.Pro47Gln	p.P47Q
Silent	SNP	onymous_var	SNV	B15_S10.vcf	c.117T>A	p.Ala39=	p.A39=
ssense_Mutat	SNP	issense_varia	SNV	B18_S13.vcf	c.172C>T	p.Pro58Ser	p.P58S
ssense_Mutat	SNP	issense_varia	SNV	B18_S13.vcf	c.140C>A	p.Pro47Gln	p.P47Q
Silent	SNP	onymous_var	SNV	B18_S13.vcf	c.117T>A	p.Ala39=	p.A39=
ssense_Mutat	SNP	issense_varia	SNV	B21_S6.vcf	c.172C>T	p.Pro58Ser	p.P58S
ssense_Mutat	SNP	issense_varia	SNV	B21_S6.vcf	c.140C>A	p.Pro47Gln	p.P47Q
Silent	SNP	onymous_var	SNV	B21_S6.vcf	c.117T>A	p.Ala39=	p.A39=
ssense_Mutat	SNP	issense_varia	SNV	B26_S12.vcf	c.172C>T	p.Pro58Ser	p.P58S
ssense_Mutat	SNP	issense_varia	SNV	B26_S12.vcf	c.140C>A	p.Pro47Gln	p.P47Q
Silent	SNP	onymous_var	SNV	B26_S12.vcf	c.117T>A	p.Ala39=	p.A39=
ssense_Mutat	SNP	issense_varia	SNV	B35_S74.vcf	c.172C>T	p.Pro58Ser	p.P58S
ssense_Mutat	SNP	issense_varia	SNV	B35_S74.vcf	c.140C>A	p.Pro47Gln	p.P47Q
Silent	SNP	onymous_var	SNV	B35_S74.vcf	c.117T>A	p.Ala39=	p.A39=
ssense_Mutat	SNP	issense_varia	SNV	B38_S18.vcf	c.172C>T	p.Pro58Ser	p.P58S
ssense_Mutat	SNP	issense_varia	SNV	B38_S18.vcf	c.140C>A	p.Pro47Gln	p.P47Q
Silent	SNP	onymous_var	SNV	B38_S18.vcf	c.117T>A	p.Ala39=	p.A39=
ssense_Mutat	SNP	issense_varia	SNV	B40_S5.vcf	c.172C>T	p.Pro58Ser	p.P58S
ssense_Mutat	SNP	issense_varia	SNV	B40_S5.vcf	c.140C>A	p.Pro47Gln	p.P47Q
Silent	SNP	onymous_var	SNV	B40_S5.vcf	c.117T>A	p.Ala39=	p.A39=
ssense_Mutat	SNP	issense_varia	SNV	B63_S3.vcf	c.172C>T	p.Pro58Ser	p.P58S
ssense_Mutat	SNP	issense_varia	SNV	B63_S3.vcf	c.140C>A	p.Pro47Gln	p.P47Q
Silent	SNP	onymous_var	SNV	B63_S3.vcf	c.117T>A	p.Ala39=	p.A39=
ssense_Mutat	SNP	issense_varia	SNV	B64_S16.vcf	c.172C>T	p.Pro58Ser	p.P58S
ssense_Mutat	SNP	issense_varia	SNV	B64_S16.vcf	c.140C>A	p.Pro47Gln	p.P47Q
Silent	SNP	onymous_var	SNV	B64_S16.vcf	c.117T>A	p.Ala39=	p.A39=
ssense_Mutat	SNP	issense_varia	SNV	B66_S15.vcf	c.172C>T	p.Pro58Ser	p.P58S
ssense_Mutat	SNP	issense_varia	SNV	B66_S15.vcf	c.140C>A	p.Pro47Gln	p.P47Q
Silent	SNP	onymous_var	SNV	B66_S15.vcf	c.117T>A	p.Ala39=	p.A39=
ssense_Mutat	SNP	issense_varia	SNV	B68_S14.vcf	c.172C>T	p.Pro58Ser	p.P58S
ssense_Mutat	SNP	issense_varia	SNV	B68_S14.vcf	c.140C>A	p.Pro47Gln	p.P47Q
Silent	SNP	onymous_var	SNV	B68_S14.vcf	c.117T>A	p.Ala39=	p.A39=
ssense_Mutat	SNP	issense_varia	SNV	B69_S11.vcf	c.172C>T	p.Pro58Ser	p.P58S
ssense_Mutat	SNP	issense_varia	SNV	B69_S11.vcf	c.140C>A	p.Pro47Gln	p.P47Q
Silent	SNP	onymous_var	SNV	B69_S11.vcf	c.117T>A	p.Ala39=	p.A39=
ssense_Mutat	SNP	issense_varia	SNV	B71_S17.vcf	c.172C>T	p.Pro58Ser	p.P58S
ssense_Mutat	SNP	issense_varia	SNV	B71_S17.vcf	c.140C>A	p.Pro47Gln	p.P47Q
Silent	SNP	onymous_var	SNV	B71_S17.vcf	c.117T>A	p.Ala39=	p.A39=
ssense_Mutat	SNP	issense_varia	SNV	B74_S4.vcf	c.172C>T	p.Pro58Ser	p.P58S
ssense_Mutat	SNP	issense_varia	SNV	B74_S4.vcf	c.140C>A	p.Pro47Gln	p.P47Q
Silent	SNP	onymous_var	SNV	B74_S4.vcf	c.117T>A	p.Ala39=	p.A39=
ssense_Mutat	SNP	issense_varia	SNV	B75_S2.vcf	c.172C>T	p.Pro58Ser	p.P58S
ssense_Mutat	SNP	issense_varia	SNV	B75_S2.vcf	c.140C>A	p.Pro47Gln	p.P47Q
Silent	SNP	onymous_var	SNV	B75_S2.vcf	c.117T>A	p.Ala39=	p.A39=
ssense_Mutat	SNP	issense_varia	SNV	B76_S1.vcf	c.172C>T	p.Pro58Ser	p.P58S
ssense_Mutat	SNP	issense_varia	SNV	B76_S1.vcf	c.140C>A	p.Pro47Gln	p.P47Q
Silent	SNP	onymous_var	SNV	B76_S1.vcf	c.117T>A	p.Ala39=	p.A39=
ssense_Mutat	SNP	issense_varia	SNV	B77_S8.vcf	c.172C>T	p.Pro58Ser	p.P58S
ssense_Mutat	SNP	issense_varia	SNV	B77_S8.vcf	c.140C>A	p.Pro47Gln	p.P47Q
Silent	SNP	onymous_var	SNV	B77_S8.vcf	c.117T>A	p.Ala39=	p.A39=
ssense_Mutat	SNP	issense_varia	SNV	B79_S7.vcf	c.172C>T	p.Pro58Ser	p.P58S

ssense_Mutat	SNP	issense_varia	SNV	B79_S7.vcf	c.140C>A	p.Pro47Gln	p.P47Q
Silent	SNP	onymous_var	SNV	B79_S7.vcf	c.117T>A	p.Ala39=	p.A39=
ssense_Mutat	SNP	issense_varia	SNV	B86_S73.vcf	c.172C>T	p.Pro58Ser	p.P58S
ssense_Mutat	SNP	issense_varia	SNV	B86_S73.vcf	c.140C>A	p.Pro47Gln	p.P47Q
Silent	SNP	onymous_var	SNV	B86_S73.vcf	c.117T>A	p.Ala39=	p.A39=
ssense_Mutat	SNP	issense_varia	SNV	B89_S75.vcf	c.172C>T	p.Pro58Ser	p.P58S
ssense_Mutat	SNP	issense_varia	SNV	B89_S75.vcf	c.140C>A	p.Pro47Gln	p.P47Q
Silent	SNP	onymous_var	SNV	B89_S75.vcf	c.117T>A	p.Ala39=	p.A39=
Silent	SNP	onymous_var	SNV	B38_S18.vcf	c.336C>T	p.Ser112=	p.S112=
Silent	SNP	onymous_var	SNV	B40_S5.vcf	c.336C>T	p.Ser112=	p.S112=

Exon_Number	Freq_in_21	Percentage
1/1	21	100
1/1	21	100
1/1	21	100
1/1	21	100
1/1	21	100
1/1	21	100
1/1	21	100
1/1	21	100
1/1	21	100
1/1	21	100
1/1	21	100
1/1	21	100
1/1	21	100
1/1	21	100
1/1	21	100
1/1	21	100
1/1	21	100
1/1	21	100
1/1	21	100
1/1	21	100
1/1	21	100
1/1	21	100
1/1	21	100
1/1	21	100
1/1	21	100
1/1	21	100
1/1	21	100
1/1	21	100
1/1	21	100
1/1	21	100
1/1	21	100
1/1	21	100
1/1	21	100
1/1	21	100
1/1	21	100
1/1	21	100
1/1	21	100
1/1	21	100
1/1	21	100
1/1	21	100
1/1	21	100
1/1	21	100
1/1	21	100
1/1	21	100
1/1	21	100
1/1	21	100
1/1	21	100
1/1	21	100
1/1	21	100
1/1	21	100
1/1	21	100
1/1	21	100
1/1	21	100
1/1	21	100
1/1	21	100

1/1	21	100
1/1	21	100
1/1	21	100
1/1	21	100
1/1	21	100
1/1	21	100
1/1	21	100
1/1	21	100
1/1	11	52.3809524
1/1	11	52.3809524
